# The Significance and Utilisation of Biomimetic and Bioinspired Strategies in the Field of Biomedical Material Engineering: The Case of Calcium Phosphat—Protein Template Constructs

**DOI:** 10.3390/ma13020327

**Published:** 2020-01-10

**Authors:** Monika Šupová

**Affiliations:** Department of Composites and Carbon Materials, Institute of Rock Structure and Mechanics, The Czech Academy of Sciences, V Holešovičkách 41, 182 09 Prague, Czech Republic; supova@irsm.cas.cz; Tel.: +420-266-009-221

**Keywords:** biomimetic, calcium phosphate, protein template

## Abstract

This review provides a summary of recent research on biomimetic and bioinspired strategies applied in the field of biomedical material engineering and focusing particularly on calcium phosphate—protein template constructs inspired by biomineralisation. A description of and discussion on the biomineralisation process is followed by a general summary of the application of the biomimetic and bioinspired strategies in the fields of biomedical material engineering and regenerative medicine. Particular attention is devoted to the description of individual peptides and proteins that serve as templates for the biomimetic mineralisation of calcium phosphate. Moreover, the review also presents a description of smart devices including delivery systems and constructs with specific functions. The paper concludes with a summary of and discussion on potential future developments in this field.

## 1. Introduction

The terms “biomimetics” and “biomimicry” derive from the ancient Greek *bios*-life and *mīmēsis*-imitation. In 1969, the term biomimetics was used by American biophysicist Otto Herbert Schmitt [1] for the title of a paper: “Some interesting and useful biomimetic transforms” on the transfer of ideas and analogues from biology to technology. By 1974, the term had found its way into the Webster’s Dictionary.

Biomimetic refers to man-made processes, substances, devices, and systems that imitate natural phenomena. Applying biomimetic logic [2], the aim is to adapt superior materials and structural designs found in nature for use in technological applications, particularly in the fields of nanotechnology, robotics, and artificial intelligence, and the medical and military sectors. Despite the fact that humans have looked to natural phenomena for inspiration for more than 3000 years, biomimetics is a relatively young field of study that considers the practical application of mechanisms and functions identified through biological scientific research in the field of engineering. Sarikaya and Aksay [3,4] divided the biomimetic strategy into two categories, i.e., biomimicking (the imitation of the design of biomaterials via the application of currently available techniques) and bio-duplication (the mastering of the molecular synthesis and processing mechanisms of biological materials so as to produce new and superior biomaterials). Although the biomimetic strategy has led to the production of a number of significant and successful devices and concepts over the past 50 years, it remains an empirical science.

The original concept behind biomimetic mineralisation focused on the description of specific synthesis protocols for the preparation of calcium phosphates [5], bioimplant coating materials [6], and mineralised composite scaffolds [7,8,9,10,11], which imitate the composition and structure of real bone. This strategy, which is based on the mutual reaction of calcium phosphates (CaP) and various organic templates, enables the use of both natural and synthetic organic compounds so as to attain complicated natural (biomimetic) or artificial (bioinspired) bioinorganic formations. Many bio-macromolecules such as proteins and polysaccharides have been reported as serving as biomimetic templates for the synthesis of calcium phosphate—organic constructs via the various functional groups present in the molecules, which are able to chelate calcium and phosphate ions. The application of proteins comprises the introduction of synthetic proteins and peptides including peptide amphiphiles or the use of natural proteins from biogenic sources that can be also obtained from biowaste. This strategy has expanded over the last decade to include a number of other applications. Crystallisation events and mineralisation phenomena play an important role in various fields of regenerative medicine, including orthopaedics and dentistry. This branch of medicine constitutes one of the most important and rapidly evolving fields in terms of exploiting the broad clinical applicability and versatility [6,12,13] of biomimetic synthesis. References in the literature to the fabrication of more sophisticated nanoconstructs based on biological supramolecular assemblies prepared for “smart” applications are becoming increasingly common. Such systems are capable of functioning as nanosensors, nanoreactors [14,15], and delivery systems [16,17] with the precise location of the intended function. Compared to other material production methods and techniques, the biomimetic approach is simple, environmentally benign, and economically feasible [18], and allows for the repair and building of hard tissues under both laboratory and in situ conditions.

The aim of this paper is to provide a review of existing literature on the topic with respect particularly to the synthesis of an organic–inorganic hybrid material based on protein–calcium phosphate as inspired by the bimineralisation process. The review focuses principally on studies published over the last 20 years or so.

The review is organised in the form of a number of sections, the first of which is the introduction. The second section summarises the current state of knowledge of biomineralisation in bone, dentin, and enamel and the third section provides a detailed description derived from the relevant literature of the application of the biomimetic strategy in the field of biomedical material engineering, including the preparation of biomimetic composite scaffolds and various sophisticated nanostructures and their applications. Finally, the summary presents concluding remarks on this active research area and discusses potential future developments.

## 2. Biomineralisation

The rapid expansion of the field of biomineralisation over the last two decades has led to the compilation of a large and expanding database of information on the various molecular participants in nucleation and crystal growth processes within organisms [19]. Living organisms are capable of inducing the crystallisation and deposition of a wide variety of minerals unlike in the case of vertebrates, the hard tissues of which primarily utilise CaP [20]. Results obtained by Jiang et al. [21] implied that biological genetic and physicochemical factors are capable of cooperatively influencing the formation of unusual complexes and hierarchical structures during apatite biomineralisation. Proteins in particular play a key role in the nucleation and crystal growth of inorganic solids in living organisms [22]. The high level of interest in vertebrate mineralisation processes has resulted in a large number of publications on the formation of apatite in bone [23], cartilage [24], enamel [25], and dentin [26] as well as pathological calcifications [27].

Bone is an example of a multi-component, hierarchically structured, mineralised nanocomposite with unique mechanical properties [12,28,29] consisting of three components: Minerals (60 wt. %), organic materials (30 wt. %), and water (10 wt. %). Ninety percent of the organic component consists of collagen, while the remainder is composed of non-collagenous proteins (NCPs), proteoglycans, and lipids.

**Figure 1:**
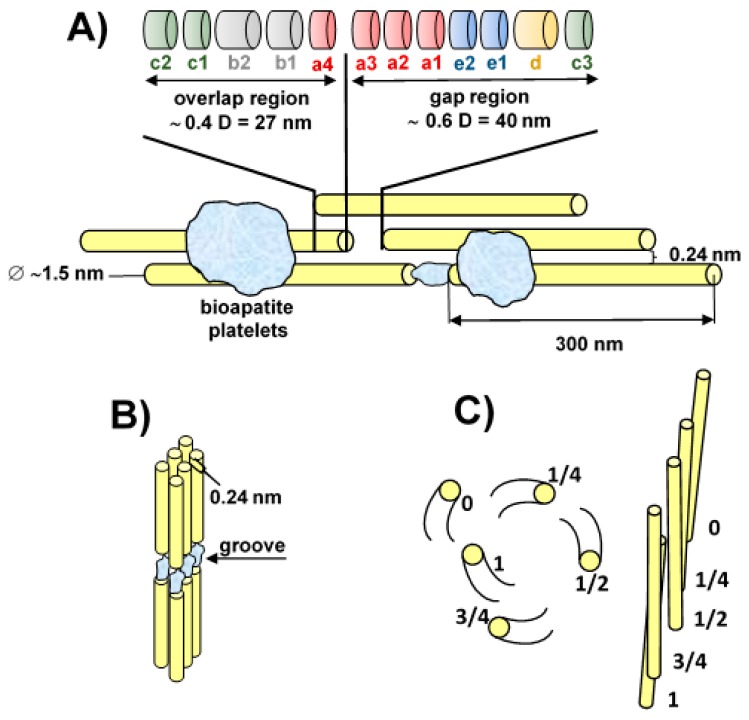
(**A**,**B**) Model for the packing of collagen molecules in bone with bioapatite platelets. Collagen molecules (straight and rod-like) are stacked end-to-end with gaps between them. (**C**) Steric model of a collagen microfibril consisting of five individual collagen molecules in a quarter-staggered arrangement labelled 0, 1/4, 1/2, 3/4, and 1 so as to indicate their relative height with respect to a horizontal plane. Collagen molecules are helically coiled along their length.

Collagen (COL) comprises two α1(I) chains and one α2(I) chain. The repetitive nature of the amino acid (AA) sequences of COL, which consists of –[Gly (glycine)-X-Y-]n where X and Y are frequently proline (Pro) and hydroxyproline (Hyp) residues, allows the individual α chains to assemble into triple helical structures referred to as tropocollagen molecules with diameters of ~1.5 nm. Collagen triple helices are stabilised via intramolecular hydrogen bonding. A 40 nm gap exists between the C and N termini of collinear tropocollagen molecules together with an adjacent 27 nm overlap zone, which are spaced every 67 nm along the fibrils, the so-called D-period (Figure 1A), which comprises 12 bands marked *a3*, *a2*, *a1*, *e2*, *e1*, *d*, and *c3* (within the hole zone) and *c2*, *c1*, *b2*, *b1*, and *a4* (within the overlap zone) [12]. They contain a series of charged amino acids (AAs), which are open so as to provide potential binding sites for the calcium and phosphate ions involved in the mineralisation process. COL gap zones have been considered to provide critical spaces for the mineralisation process. They appear to constitute early stage of bioapatite (BAP) nucleation sites and extend into the overlap zones during the later stages of intrafibrillar mineralisation [30]. Gaps (each with a thickness of ~1.5 nm) form a zone that can be traced across each fibril; the gaps are aligned so as to form grooves (Figure 1B). However, in reality, COL molecules are not rod-like but are right-handedly helically twisted in a discontinuous manner along their lengths and are bound together at specific locations to form microfibrils, which can be defined as an assembly of five individual COL molecules arranged in parallel and quarter-staggered along their axial direction (Figure 1C).

While the bone mineral (BAP) is idealised as hydroxyapatite (Ca_10_(PO_4_)_6_(OH)_2_), it is associated with minor groups and elements (e.g., CO_3_^2−^, HPO_4_^2−^, Na^+^, Mg^2+^) and trace elements (e.g., Sr^2+^, K^+^, Cl^−^ and F^−^), some of them at the ppm level, which play a vital role with respect to bone metabolism. Individual crystals of bone mineral are plate-shaped that develop on the (100) face of the apatite crystal lattice and have dimensions of <1 nm thick and 30–40 nm in diameter [12]. These nanocrystals are embedded within COL fibrils with their *c*-axes arranged roughly parallel to the long axis of the fibrils and, together, they create an organic–inorganic core-shell structure [31].

The formation of the plate-like crystals is still not fully understood; one possible explanation for such mineral morphology in bone is that crystal growth occurs via an octacalcium phosphate (OCP) intermediate that has almost the same crystal structure as hydroxyapatite (HA) and that it contains a hydrated layer that may be responsible for the plate-shaped crystals observed in natural bone [32]. Others discussed the CaP phase, as an intermediate phase in the in vitro formation of OCP and HA, is brushite (CaHPO_4_·2H_2_O, DCPD) [33]. A question that continues to arouse controversy with respect to bone mineralisation theories, concerns whether this mineralisation pathway commences with an amorphous calcium phosphate (ACP) [34] such as a transient precursor.

With a view to enhancing the understanding of intrafibrillar mineralisation (deposition inside COL microfibril), various in vitro models have been proposed based on three main principals [12]:

1. Electrostatic interaction,
2. Capillary forces,
3. Size-exclusion models.

1. It is generally accepted that vertebrate mineralisation is closely related to the multiple electrostatic interactions between charged side chains of proteins in the extracellular matrix (ECM) [35] and free Ca^2+^ or PO_4_^3−^ ions. The active role of COL type I in the direct nucleation of CaP crystals has been the subject of heated debate for several decades. Some studies suggested that the COL itself is not capable of initiating biomineralisation, acting rather as a passive store for the resettlement of apatite crystallites through NCP interactions [36]. Conversely, other reports suggested that collagen does provide a heterogenous nucleation template without the need of NCPs via electrostatic interactions between apatite and COL [37]. The carboxylic groups (–COO^−^) of glutamic (Glu), aspartic (Asp), and glucuronic (Glc) acid sidechains attract and bind two Ca^2+^ ions from solution and further attract PO_4_^3−^ ions so as to form a connective CaP network with consequential nucleation [38,39]. A large number of NCPs and phosphorylated modifications thereof [40,41] are strongly involved in the matrix vesicle-mediated mineralisation process and play essential roles in the regulation of bone biomineralisation, such as via the initiation of the formation of ACP, apatite nucleation, and crystal growth, as well as via inhibition [42]. It is generally assumed that NCPs regulate solution crystal growth via some sort of ‘epitaxial’ relationship between specific crystallographic faces and specific protein conformers [43]. Polyanionic electrolytes, such as poly (aspartic acid) (PAsp) [38,44] and poly(L-arginine) (PA) [45] have been used (via the mimicking of NCP functional groups) for the modelling of the mineralisation process in vitro. Previous studies proved the significant effect of inorganic additives present in biological fluids such as Mg^2+^ [46] Cl^−^ and NO_3_^−^ [47] on CaP formation and morphology. However, most of the in vitro studies on apatite/AA interactions performed to date were conducted in environments that differed significantly from in vivo conditions, under which several other agents such as mineralisation inhibitors and cells are involved in apatite precipitation [48].

2. Capillary force theory allows for the effective clarification of intrafibrillar mineralisation by suggesting that CaP crystals derive from a liquid-phase amorphous precursor, which is drawn into gaps and groove (Figure 1A,B) zones by means of capillary forces. Olszta et al. [43] were the first to put forward the polymer-induced liquid precursor (PILP) concept, which suggests that the charged polymer acts as a process-directing agent, by which conventional solution is converted into a precursor that further crystallises into a more thermodynamically stable phase, leaving COL fibrils embedded with CaP nanocrystals.

3. The size-exclusion theory supposes that COL fibrils are also able to act as a regulator and control mechanism with respect to determining the molecular weight of the protein that is able to freely diffuse into the inner spaces of collagen fibrils. It has been postulated [49] that proteins with molecular weights lower than 6 kDa are able to freely diffuse into the inner spaces of COL fibrils, whereas molecules with weights of over 40 kDa protein are excluded.

The mechanisms of CaP mineralisation in differing biological matrices have not yet been fully identified due to the large number of factors that influence prenucleation, nucleation, growth, and the structure and composition of the polymers that participate in the mineralisation process and their mutual interactions. In addition to novel laboratory and experimental technologies, computer simulations are able to provide particularly useful insight into the mineralisation process [50]. The development of such simulation models contributes to both the improvement of material synthesis strategies and the fundamental understanding of natural processes such as biomineralisation [51].

## 3. The Biomimetic Strategy in Biomedical Material Engineering

Biomineralisation provides an ideal model for bioinspired fabrication purposes. Living organisms control crystal nucleation and growth using organic interfaces as templates. It has been shown that some chemical groups of proteins, as well as the tri-dimensional matrix in which calcification occurs, play a fundamental role in the nucleation and growth of apatite. Over the last two decades particularly, scientists have turned to nature for assistance in learning how to design biomimetic biomaterials inspired by the hierarchical complex structure of bone and other natural mineralised tissues, and to regulate the biomineralisation process on biomaterial substrates so as to enhance the osteoconductive properties of implantable devices.

### 3.1. Biomimetic Composites and Scaffolds for Bone Regeneration Purposes

Several research groups have directed their efforts towards the development of composite materials and engineered tissues with concern to bone and osteochondral regeneration [52] employing several approaches [53]. A number of researchers have mixed CaPs with organic matrices to form particle-reinforced scaffolds that mimic the composition but not the structure of bone. Bone is an interpenetrating phase composite and, since particulate composite scaffolds embody higher inhomogeneity, they do not possess either bone-like mechanical or biological properties. The simple mixing of individual components is unable to result in the same interaction between organic and inorganic phases obtainable via mineralisation in situ. Many bio-macromolecules such as collagen, gelatin, and polysaccharides have been reported to serve as biomimetic templates for the synthesis of nano-CaP via the various functional groups present in the molecules, which are able to chelate Ca^2+^ ions and form hydrogen bonds with protonated PO_4_^3−^ and H_2_O on the surface of the mineral.

#### 3.1.1. Collagen and Gelatin/CaP Systems

Collagen (COL) is a well-known protein component that possesses the capacity to mineralise in a variety of vertebrate tissues. Gelatin (GEL), a protein that is obtained through the hydrolysis of COL, the primary protein in bone, is inexpensive, widely available, and frequently used in the food industry. In their mineralised forms, both have the potential for use as biomimetic materials with respect to a variety of applications and they have been employed to date in a wide range of research studies concerning tissue engineering.

The fundamental aspects of biomineralisation may also prove important in terms of proposing new methodologies for the development of bioactive tri-dimensional templates with hierarchically ordered textures [54,55,56,57,58]. With respect to the healing of bone defects and tissue engineering matrices, scaffolds featuring porous interconnected networks with sufficient mechanical strength are required that can easily be pre-seeded with cells in the laboratory and invaded by tissue following implantation. The preparation of scaffolds that mimic the organisation of the bone requires the creation of an environment that closely resembles the natural bone ECM. Two principle approaches have been reported to date concerning the preparation of COL(GEL)/CaP matrix composites:

(1) The direct, in-situ mineralisation of COL(GEL) [56,58,59,60,61,62,63,64,65,66,67,68],
(2) The biomimetic deposition of CaPs on pre-arranged COL(GEL) scaffolds [41,69,70,71,72,73] from Ca^2+^ and PO_4_^3−^ ion-containing solutions, e.g., simulated body fluids.

Several papers performed a particularly interesting study on the dependency of the reaction products on the amount of GEL, in which they proved that lower GEL concentrations lead to smaller plate-like particles (~tens of nm) compared to higher concentrations that lead to the development of large foils (~hundreds of nm). Moreover, the prestructuring components, meaning which ions first reacted with the GEL, i.e., PO_4_^3−^ or Ca^2+^, was found to strongly influence the composition of the final product [74,75]. Further factors include concentration of GEL, pH [64,65,68,76,77,78,79,80] and temperature of coprecipitation [81].

COL scaffolds and composites have been subjected to mineralisation via the PILP process, involving the use of an electrolyte that forms a fluidic ACP precursor. For illustrative purposes, poly(acrylic acid) (PAA) [82,83,84] and tripolyphosphate (TPP) [83] were applied to simulate the N- and C-terminal domains of NCP and DMP-1, respectively. These “soft templates” can be used in the “bottom-up” approach for the fabrication of hierarchically biomimetic COL/CaP scaffolds [85]. Thula et al. [86] applied PILP for the investigation of the effectiveness of this process in the mineralisation of dense COL substrates. The various conditions included polymer molecular weight, substrate dimensions, and the mineralisation time were applied. Mineral penetration depths of up to 100 µm were attained via the PILP process compared to the conventional crystallisation process via which no penetration, i.e., only surface precipitates, were observed. A study by Antebi et al. [87] combined a unique dynamic flow with PILP for the fabrication of a porous COL/CaP composite. The dynamic flow imitated the physiological extracellular fluid flow containing the mineralisation ions, while the PILP method facilitated the intrafibrillar mineralisation process. Wingender et al. [88] improved upon the PILP technique via molecular crowding, a procedure that is used for the densification of acidic COL type-I solutions beyond the critical concentration (~90 mg/mL). This method resulting in hierarchical composites with a high degree of mineralisation and a composition corresponding to that of lamellar bone.

**Figure 2:**
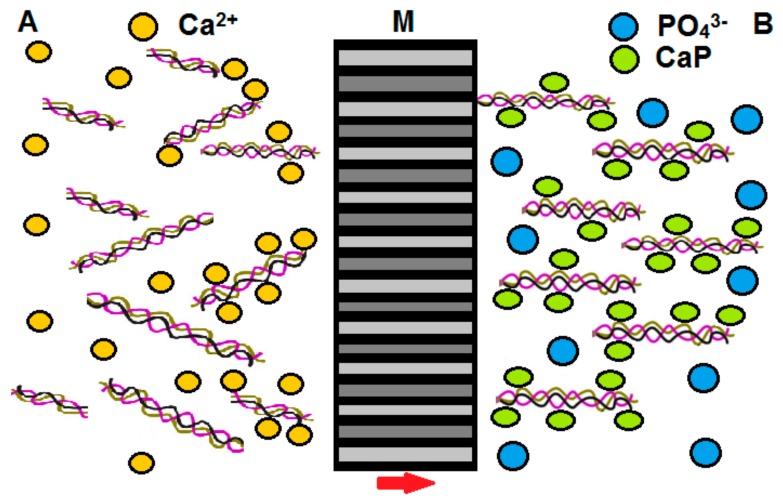
Experimental setup based on a nanoporous membrane (M) for the formation of mineralised COL fibrils as proposed by Maas et al. [89]. (**A**) Solution containing Ca^2+^ cations (yellow) and monomolecular tropocollagen. (**B**) Solution containing PO_4_^3−^ anions (blue) and the formation of amorphous CaP (green) together with the self-assembly of collagen fibrils in the direction of the flow of the feed solution (red arrow).

In some cases, special additional procedures have been applied to simple CaP precipitation. Tomomatsu et al. [63] employed enzymatic mineralisation with an alkaline phosphatase (ALP) to catalyse the hydrolysis of water-soluble phosphate esters in the presence of Ca^2+^ ions. ALP is prone to acting on organic phosphates so as to generate phosphate ions, which are subsequently able to react with Ca^2+^ ions arranged in an orderly way on COL molecules. Wang and Liu [62] applied the microwave irradiation process during precipitation whereupon the reaction between the Ca^2+^ and PO_4_^3−^ ions accelerated significantly accompanied by a reduction in the particle size. Maas et al. [89] presented a particularly interesting method for the preparation of mineralised COL fibrils (Figure 2) based on a nanoporous polycarbonate track-etched membrane (M) that separated two liquids, i.e., a feed solution (A-containing both Ca^2+^ cations and monomolecular tropocollagen) and a receiver solution (B-containing PO_4_^3−^ anions leading to the formation of mineralised nanofibres at the exit of the pores). While this approach had been used previously for the preparation of nanoparticles, Maas et al. expanded the method so as to produce fibrils accompanied by the control of the size of the nanofibre diameters.

A porous and interconnected structure, together with the consequent transformation of nanocomposites into a hierarchical porous scaffold, is usually attained via the application of a a freeze-drying technique that does not require a solid porogen. Shen et al. [56] prepared a COL/CaP hierarchical porous three-dimensional (3D) scaffold with well-controlled interconnected porosity. The composite exhibited a well-developed macropore structure with a pore size of around 50–100 µm and sub-micropores with a pore size of 1–5 µm and contributed to osteogenesis. Xia et al. [55] created unidirectional aligned macro-pores with a size of 64 to 344 μm by controlling the freezing rate and direction. Pore diameters of around 200 μm have been shown to be highly suitable in terms of homogeneous cell seeding [60].

The mineralisation of COL/GEL matrices significantly enhances the mechanical properties of the scaffolds since the CaP crystallites in the COL/GEL fibrils restrict the deformation of the COL fibril network [62,66]. Xia et al. [55] proved that a multi-level mineralised lamellar COL structure resulted in a 12-fold increase in the Young’s modulus and a two-fold increase in the compression modulus along the aligned direction compared to a scaffold of the same composition with an isotropic equiaxed pore structure. Moreover, Liu et al. [78] successfully prepared a multi-level hierarchically ordered CaP/GEL composite with a modulus in the perpendicular direction of the HA *c*-axis of ~26 GPa and a hardness of ~0.9 GPa, which correlates well with that of human bone. Yokoyama et al. [60] prepared COL/CaP scaffolds with high elasticity in the wet state, thus offering the potential for cell culture under cyclic mechanical loading; in addition, the elastic properties enabled the easy handling and adaptable geometry of the defects. Suchý et al. [90] proved that hydration exerts a major statistically significant effect on the mechanical behaviour of polymer/CaP scaffolds because it more precisely simulates the real environment for which such materials are designed.

COL and GEL embody poor mechanical properties, high swelling rates in aqueous environments, low structural stability, and a low level of resistance to enzymatic degradation. Thus, cross-linking methods were developed aimed at both improving the material mechanical properties and at retarding the biodegradation rate of COL-based biomaterials.

Chemical cross-linking is achieved principally via covalent amine/imine linkage. The most popular cross-linking method employed involves the application of a carbodiimide, especially 1-ethyl-3-(3-dimethyl aminopropyl) carbodiimide hydrochloride (EDC) [60]. EDC is considered to be both efficient and relatively harmless since the combination of carbodiimide with N-hydroxysuccinimide (NHS) [71,72] results in the formation of amides between COL molecules and all the excess residues can be removed. One of the main reasons for the popularity of EDC/NHS treatment lies in its higher degree of compatibility than that of commonly employed bifunctional cross-linkers such as glutaraldehyde (GA) [61,91].

The cross-linking process can be applied:

(1) In one step together with COL/GEL mineralisation [61,91],
(2) Prior to mineralisation [41,70,71,72],
(3) Following mineralisation [60,66,92,93,94].

Only a limited number of studies have addressed the influence of the cross-linking process on mineralisation. Chang et al. performed the directional assembly of HA/COL [91] and HA/GEL [93] fibrils induced by GA cross-linking. They discovered that cross-linking induced the shortening of the distances between the HA/COL fibrils within the critical length, thus allowing for a greater number of Ca^2+^ ions on the HA to bind with the COL molecule R–COO^−^ ions. Li et al. [71] also proved that COL cross-linking by EDC/NHS enhanced the mineralisation process. They suggested that the cross-links in the COL films, rather than preventing, in fact promote mineralisation through their “propping open” the intermolecular spaces and minimising the exclusive volume of COL fibrils, which, in turn, facilitates the infiltration of polyAsp that mimic the functional groups of NCPs. Narasimha Raghavan et al. [70] cross-linked fish scale collagen with 3-aminopropyl triethoxysilane. Since silanol groups (Si–OH) are acidic, they interact with calcium ions and, thus, act as initial *loci* for the heterogenous nucleation of HA. Zhou et al. [95] proved that the application of UV irradiation to GEL methacrylate hydrogel suppressed mineralisation via the consumption of the free charges, which inhibited the infiltration of precursor ions and, thus, resulted in a decrease in mineral deposition.

However, since synthetic cross-links differ substantially from chemical cross-links in bone COL, it is difficult to propose definitive conclusions regarding cross-linking mineralisation capability in bone from the afore-mentioned studies.

While BAP is idealised as HA, it is substituted with minor groups and elements, as described in Section 2. Substituted CaP can be synthesized via wet chemical co-precipitation. Synthetically substituted CaP can be tailored by means of the substitution level of the precursors thus allowing for the preparation of a final product with bespoke properties [96]. Carbonates (CO_3_^2−^) constitute the major substituent in biological apatite. Several papers have described the preparation of carbonated HA/COL composites [72,91,97,98], while labile carbonates already exist in such solutions in the form of CO_3_^2−^ or HCO_3_^−^ anions. Carbon dioxide dissolved in the solution during the precipitation procedure may also have served as a carbonate source in the apatite samples and have naturally co-precipitated with the phosphate anions [99,100]. Magnesium constitutes one of the principal cations found in young and newly formed bone. Magnesium-substituted HA directly precipitated on COL fibres have been prepared by Minardi et al. [101] and by Sader et al. [92]. Tampieri et al. [102] prepared multi-substituted HA, e.g., magnesium-carbonate-HA and silicon-magnesium-HA via nucleation into and onto COL fibres consisting of a true amorphous phase. While Sr^2+^ cations, one of the foreign ions present in bioapatite, are able to diffuse into the walls of Haversian capillaries, replace Ca^2+^, and enhance bone volume, which has led to its use in the prevention and treatment of osteoporosis. Huang et al. [100] incorporated Sr^2+^ together with CO_3_^2−^ anions into HA by means of biomineralisation. Their results revealed that the incorporation of Sr^2+^ into HA did not influence the organisation of apatite crystals or COL fibrils.

Finally, a number of studies mentioned in this chapter also considered biocompatibility via cellular in vitro testing using the MC3T3-E1 preosteoblast cell line [55,63], L-929 fibroblasts [69,72], osteoblast SAOS2 cells [92], and human bone marrow-derived mesenchymal stem cells [65,101]. The osteoinduction of materials was proved via in vivo testing. Yokoyama et al. [60] implanted materials into the subcutaneous tissue and bone defects introduced into the femur of rats, with harvesting with the surrounding tissues lasting up to 12 weeks following surgery. The materials implanted in the subcutaneous tissue were resorbed at 8 weeks, and new bone formation was observed on the surface of the materials at 1 week. Minardi et al. [101] using an ectopic model in a rabbit proved that the scaffold produced a large volume of trabecular bone after only two weeks, which was followed by the increased formation of mature cortical bone.

The structures of the templates play a vital role in controlling the morphologies of inorganic crystals [103]. Fibrous templates i.e., COL, lead to a more needle-like HA, while globose templates i.e., chitosan, result in sheet-like HA. Most studies have focused on the use of just one organic template, while natural bone provides an example of a multi-template composite. Inspired by such a mechanism, it is possible to apply a strategy based on multi-template co-assembly for the fabrication of bone-like material. Various natural macromolecules based on polysaccharides e.g., chitosan (Chi) [67,104,105,106,107,108], glycosaminoglycans e.g., chondroitin sulphate (ChS) [109,110,111,112], hyaluronic acid (HYA) [113], and various proteins such as silk fibroin (SF) [114,115] and phosvitin [41] have been used in combination with COL. Conversely, GEL is more commonly used in combination with synthetic polymers e.g., PAA [73,116], metacrylic anhydride [95], and polyvinyl alcohol [117].

The simultaneous application of different templates in one system provides a greater number of nucleation sites and active centres than single-template systems; moreover, the former influences not only the orientation, size, and shape of the CaP crystals, but also results in the homogeneous distribution of nano CaP crystals [106,107,114]. Yang et al. [110] proved that macromolecular configurations of COL and ChS fibrils enabled the formation of directionally petal-like HA crystals similar to those founded in bone, while the precipitation of HA in a COL/Chi mixture at a ratio of 1/1 (*w*/*w*) by Teng et al. [107] resulted in the formation of needle-like nano-particles as did a COL/SF bitemplate [114]. Wang et al. [118] applied an anionic derivate of Chi, carboxymethyl chitosan (CMC) enriched in carboxyl groups, that is able to imitate the function of the NCPs and the dentin matrix protein. A previous study by the same team [119] demonstrated that CMC is able to stabilise ACP so as to form liquid-phase nanocomplexes of CMC/ACP, which are able to assist in the intrafibrillar mineralisation of COL and, thereby, facilitate the re-mineralisation of demineralised dentine. The most commonly used approach to the fabrication of COL/Chi scaffolds involves direct mixing under acidic conditions that, however, are not favourable for the assembly of COL fibrils. Therefore, COL/Chi scaffolds can be fabricated via a co-fibrillogenesis step at physiological pH at which the potential electrostatic interactions between anionic COL and cationic Chi result in the formation of COL/Chi fibrillar complexes dependent on the COL/Chi relative ratio and the actual pH. However, if the biomimetic precipitation of CaP is applied in such a system, it may exert a negative influence on the homogeneity of the resulting nanocomposites. Therefore, Luo et al. [105] developed a pH-triggered synthesis strategy at a low temperature (4 °C) aimed at initiating COL self-assembly, Chi deprotonation, and the precipitation of CaP via ammonia diffusion into an acidic homogeneous solution. Huang et al. [120,121,122] designed an injectable hydrogel scaffold as a result of the ability of a Chi/HA/COL solution to rapidly form a stable gel at body temperature. Both COL and GEL are easily modifiable by glucuronic acid (Glc), which exerts remarkable effects on biomineralisation by enhancing the number of accessible carboxyl groups and, therefore, modifying the support for the formation of HA, while unmodified COL yields the formation of OCP, DCPD, and DCP—CaP phases with a lower Ca/P ratio than that of HA [123]. Carboxymethylation has been found to enhance mineralisation capacity [124] and the application of an electric field to accelerate CaP formation [39,59].

Kanungo et al. [112] fabricated a ChS/COL/CaP scaffold by means of freeze-drying and determined that the mechanical properties (Young’s modulus) for a 50 wt. % mineralised scaffold was roughly 780 kPa. Their attempt to increase the mechanical properties of the scaffold by increasing the mineralisation from 50 to 75 wt. % was unsuccessful due to defects in the more mineralised scaffold. Further, in [109], they described a new technique for enhancing the mechanical properties by increasing the relative density of the scaffolds via an increase in the volume fraction of solids in the slurry employing vacuum-filtration. The Young’s modulus in the dry state increased from 780 to 6500 kPa and in the hydrated state from 6.44 to 34.8 kPa.

#### 3.1.2. Other Protein/CaP Systems

Nanoscale-structured materials can be fabricated via the spontaneous organisation of self-assembling proteins so as to construct hierarchically organised nanomaterials. Various natural and synthetic proteins and polypeptides can be specifically designed as building blocks that incorporate molecular recognition features that provide a physiological environment for cells both in vitro and in vivo [125].

Nevertheless, COL/GEL, as natural polymers, are characterised by batch-to-batch variability and local inhomogeneities and, as products derived from animal tissues, they may display immunological reactions or contamination by pathogenic substances. However, these limitations can be overcome by the introduction of synthetic proteins and peptides. Peptides consist of AA sequences with less complex functionality than proteins, and their synthesis with the desired AA sequences can be accomplished via the use of chemical and genetic engineering techniques, which are significantly simpler to apply than is the synthesis of proteins [126]. Block copolymer-peptide conjugates can be used with varying charges on the protein chains (nonionic, anionic, and zwitterionic) in order to influence the mineralisation process [127,128].

##### Synthetic Peptide/CaP Systems

COL mimetic peptides [129], such as those derived from recombinant COL type I (RCP) have been enriched with the arginine (Arg)-Gly-Asp acid sequence (RGD) [53,130,131], which is found in numerous cell-surface proteins, including integrins, which, in turn, act as receptors for cell adhesion molecules. Synthetic COL models that share the structural features of native COL are commercially available. Ramírez-Rodríguez et al. [130,131] proved the synergistic effect of both RCP and Mg^2+^ in stabilising the ACP precursor. Other attractive recombinamers can be derived from elastin (an ECM protein that provides tissues and organs with elasticity). Elastin-like recombinamers (ELRs) consist of biosynthetic polypeptides based on a repeating pentapeptide sequence derived from tropoelastin and embody inverse transition temperatures (Tt) that allow them to transit between the soluble form (T < Tt) and insoluble aggregations (T > Tt). Depending on the exact sequences, ELRs can be designed so as to be water soluble at room temperature and to precipitate at body temperature, e.g., in a bone defect over an extended period of time. Li et al. [132,133] synthesised thermo-responsive hydrogels using ELRs as mineralisation templates applying PILP mineralisation, and successfully prepared a composite with an elastic modulus and hardness of the same order of magnitude as those of natural hard tissues. Prieto et al. [134] employed different ELRs modified with the SNA15 domain of statherin, a protein in humans that prevents the precipitation of CaP in saliva, thus maintaining both the high calcium level necessary for the remineralisation of tooth enamel and the high phosphate levels required for buffering. The N-terminal 15 amino acids of statherin (SN15) and its analogue (SNA15) exhibit a higher affinity for HA than does the whole statherin molecule due to their negative charge density and helical conformation. Chen et al. [135] proved that HA precipitated in-situ in an SN15 environment were nanosized together with a significant increase in the amount of peptide adsorbed on the surface of the HA.

Peptide-based self-assembly provides a further promising approach concerning the design of biomineralisation templates. This strategy employs peptide amphiphiles (PAs) that are able to self-assemble into structures that include high-aspect ratio nanofibres [136,137]. PA possesses three regions, i.e., a hydrophobic tail, a region of β-sheet-forming AAs, and a peptide epitope that allows for the solubility of the molecule in water and the performance of its biological function via its interaction with living systems. The supramolecular morphology of PA nanofibres can be controlled through the primary peptide sequence via the regulation of intermolecular forces, i.e., hydrogen bonding strength, peptide chirality, and the distribution of the electrostatic charge. A pilot study by Hartgerink et al. [138] resulted in a particularly well-designed PA; the peptide had an alkyl tail, four cysteine residues for the formation of disulphide bonds, three Gly residues that functioned as a flexible linker, a phosphorylated serine (Ser) residue for interaction with Ca^2+^ ions, and, finally, RGD-a cell-adhesion ligand. Newcomb et al. [139] studied the ability of PA assemblies to guide HA nucleation by varying the overall charge and propensity for the β-sheet hydrogen bonding of the PA molecule, proving that the nanofibre surfaces, functionally enriched in concentrated arrays of acidic and phosphorylated residues, were important with respect to bone mineralisation [140]. For example, Mata et al. [141] proved that enhanced bone regeneration is linked to the presence of phosphorylated Ser residues that acted to accelerate the HA crystallisation kinetics in a non-healing rat femoral defect [39].

Self-assembled hydrogels that mechanically resemble the native ECM present an attractive alternative for the in-situ formation of tissue scaffolds. Gungormus et al. [142] described the development of a self-assembling peptide hydrogel that is capable of directing the mineralisation of CaPs. The MDG1 (mineral directing gelator) 27 residue peptide undergoes triggered folding to form a non-symmetrical β-hairpin that self-assembles in response to increasing ionic strength in solution so as to yield a mechanically rigid hydrogel. The C-terminal portion of MDG1 contains a heptapeptide (MLPHHGA) that has been proved to be able to control the mineralisation process.

However, since the mechanical properties of protein/CaP systems are insufficient for the support of major load-bearing devices, combinations of CaP-based nanocomposites and other materials are increasingly being developed and applied. A number of studies have suggested that carbon nanotubes (CNTs) are also able to improve the properties of biomaterials. High aspect ratios, strength stiffness, high tensile strength, excellent flexibility, and low density render CNTs ideal for the production of lightweight high-strength functional materials that resemble bone. Aimed at improving the degree of biocompatibility, Wei et al. [143] biomimetically precipitated flake-like microcrystals of HA upon functionalised multi-walled CNTs with the Z-Gly N-succinimidyl ester bi-functional linker. Graphene oxide (GO) is an amphiphile scaffold with a largely hydrophobic basal plane and hydrophilic edges that has the potential to act as a nanoscale reinforcement material for bone tissue engineering purposes due to its high mechanical strength, large specific surface area (SSA ~ 2600 m^2^/g), and good biocompatibility qualities. Moreover, reactive oxygen functional groups have the potential to provide reactive sites for functionalisation. For example, Liu et al. [144] reported the modification of GO by means of GEL so as to mimic the charged proteins present in the ECM during bone formation. Wang et al. [145] demonstrated a simple strategy for the creation of self-assembled peptide nanofibres (PNFs) with head and tail motifs (AEAKAEAK) and a centre motif (YWYAF) that possesses the ability to bind with carbon surfaces. The PNFs promoted the formation of HA nanocrystals along their axes during short-term incubation (several hours) and the GO nanosheet mediated the formation of an HA micro-sphere following long-term mineralisation (>1 day).

##### Natural Protein/CaP Systems

With the increasing demand for innovation in the field of biomaterials, the use of natural proteins from animal and botanical sources is increasingly being subjected to experimentation.

**Figure 3:**
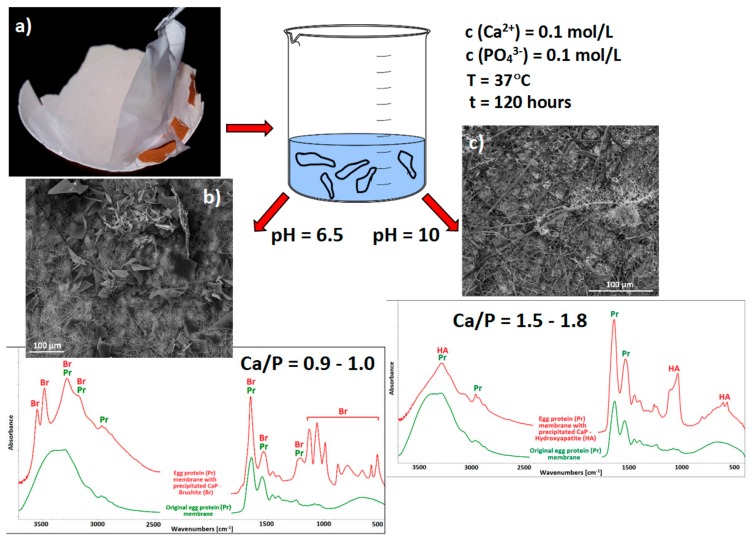
(**a**) Eggshell biomembrane used as a template for CaP precipitation under physiological conditions applying two different pH values. SEM and FTIR analysis (**b**) for pH = 6.5 and (**c**) for pH = 10. Pr = protein; HA = hydroxyapatite; Br = brushite.

Yoh et al. [146] employed a fibrin gel for fluoroaptite (FA) precipitation purposes in the preparation of a biocomposite for hard tissue applications. Fibrin is both biocompatible and biodegradable and can be obtained from peripheral blood. The team proved that fluoride (F^−^) anions contributed to the stability, enhanced the crystallinity, and inhibited the deformation and hydration of the resulting crystals. An increment in the F^−^ ion concentration led to the alteration of the mineral from OCP/HA to HA/FA in a concentration range of 2–500 ppm of F^−^ ions. Greater concentrations resulted in the preferential formation of CaF_2_. Due to the high cost and difficulty of extraction of certain proteins from organisms, new alternatives are gradually being introduced. Zhao et al. [147] employed ovalbumin (OVA) extracted from egg white as a natural biosurfactant for the preparation of large HA/OVA composite particles (several mm in length and diameter). OVA is a protein that exhibits a stable structure and biocompatibility; it is abundantly available, involves low production costs, and can be modified so as to obtain a variety of chemical, physical, and biological properties. A different biomimetic strategy employed eggshell biomembrane (EGSM) as a crystalliser for the precipitation of CaP and the growth of crystals [148,149,150]. Moreover, the influences of various factors such as temperature, pH, and holding time on the morphology and crystallinity of the agglomerates have been studied in detail by Zhang et al. [149] via the application of EGSM as a semi-permeable septum that divides Ca^2+^ and PO_4_^3−^ ion-containing solutions. Our research group, inspired by this research, attempted to simply immerse EGSM in a solution containing both Ca^2+^ and PO_4_^3−^ ions under physiological conditions for five days and considering two different pH value variations (6.5 and 10) (Figure 3). Both the morphologies and crystal structures of the agglomerates were found to be strongly influenced by the pH values. The pH value of 6.5 favoured the precipitation of flower-like DCPD, which proved to be brushite agglomerates following SEM and FTIR analysis (Figure 3b). The higher pH value of 10 resulted in a higher driving force for the formation of HA (Figure 3c) with the decreasing size of the precipitates, as was demonstrated by Zhang et al. [149].

Further inspiration has been provided by the adhesion of mussels to ships and rocks under wet conditions. The adhesive proteins involved contain principally dihydroxyphenylalanine (DOPA) and lysine. Dopamine (DA) contains the same functional group as that of the DOPA residue side chain, which endows DA with strong adhesive properties with respect to a wide range of materials due to its self-polymerisation in a weakly alkaline aqueous environment (pH = 8.5) so as to form polydoapmine (PDA) [151]. Since PDA is not a real protein, rather a biogenic amine (an amino acid derivate), it is mentioned and described in this review paper. Ryu et al. [152] reported a universal biomineralisation route that enables the directional *c*-axis growth of HA crystals upon a PDA film adhered to various scaffolds. Liu et al. [153] proved that PDA promoted rapid mineralisation when combined with a CaP cement so as to form a layer of nanoscale bone-like CaP. Gao et al. [154], via the introduction of a preparation of PDA-CaP into a polycaprolactone (PCL) matrix by means of co-electrospinning, proved that composite nanofibres prepared in such a way exhibit favourable cytocompatibility using human mesenchymal stem cells (hMSCs) at a given PDA-CaP concentration in composite nanofibres of up to 10 wt. %. Kim and Park [155] prepared carbonated HA (CHA) using PDA mineralised with CaCO_3_ (vaterite) followed by chemical conversion in SBF. They discovered that PDA stabilised the vaterite phase and also influenced the level of conversion to CHA. Deng et al. [156] proved that PDA template-mediated CaP crystals prepared via a biomimetic strategy exhibited higher cell proliferation, spreading, and alkaline phosphatase activity in vitro as well as higher levels of bioactivity and bone formation in vivo (rabbit calvarial defects) than did PDA-coated CaP crystals.

Silk fibroin (SF) extracted from silk cocoons [157,158,159,160] provides a further promising material with respect to biomimetic CaP precipitation. SF is a linear polypeptide comprising 17 amino acids with a general essential amino acid sequence of (Gly-Ser-Gly-alanine (Ala)-Gly-Ala)_n_ that exhibits good oxygen-dissolved permeability in the wet state, as does human skin, together with high resistance to water and strong mechanical properties, thus rendering it a promising potential material for use in wound dressings. SF embodies two kinds of crystalline conformations, i.e., a α-helix and a β-sheet, the latter of which is superior to the α-helix in terms of the above-mentioned properties. The conformational transition from α-helix to β-sheet form can be achieved via a number of treatments, e.g., heating or immersion in polar solvents. Wang et al. [161] proved that SF modification with an alkali solution or an enzyme (Proteinase K) improved the interface between HA and the SF matrix. The disintegration of SF into a large number of small fragments resulted in an increase in the contact number density, thus intensifying the chemical interactions between the HA and the SF matrix and, consequently, strengthening the degree of interfacial bonding as evidenced by enhanced micro-hardness. Yang et al. [162] proved that the nucleation of HA was controlled by the molecular self-assembly of SF with respect to the imitation of organic–inorganic hybrid tissue. Most studies on the subject have reported an additional requirement concerning either the pre-exposure of SF or its association with calcium and phosphate solutions so as to promote HA nucleation and growth [163,164,165,166,167,168]. However, intrinsic material properties such as surface roughness, geometry, specific surface area, tortuosity, and secondary conformation may influence the mineralisation process. Other questions relate to the processing parameters during dissolution, which may strongly influence the native structure of SF chains and their tendency to re-assemble the necessary β-sheet conformation. Finally, the inherent inert nature of SF chains must be considered. In order to gain an understanding of the mechanism of the template-induced nucleation and growth of HA nanocrystals on SF, Midha et al. [169] studied the molecular interactions of HA with native versus regenerated SF fabricated in different morphologies. SF also offers a very attractive option in terms of the preparation of a biomimetically precipitated SF/CaP composite in the form of nanofibres prepared by means of electrospinning [170,171] and in the form of a 3D hydrogel [172,173,174]. Utku et al. [175] were the first to employ the electrochemical deposition of carbonated HA on an SF scaffold as an alternative to the classic biomimetic method. The nature and origin of the silk significantly affects the quality of the scaffold, i.e., it has been proved that scaffolds constructed from eri (*Philosamia ricini*) or tasar (*Antheraea mylitta*) silk promote bone regeneration more effectively, and are more hydrophilic and mechanically stronger, than those fabricated from silk fibroin from *Bombyx mori* [176]. Tussah silk (TSF) has a higher Ala content than domestic silk; in addition, it contains an Arg-Gly-Asp motif, which is thought to both function as a biological recognition signal and to promote cell adhesion; thus, TSF/HA biocomposites represent a novel set of biomaterials with the potential for application as scaffolds in the fields of tissue engineering and bone regeneration [171].

In general, the simultaneous application of different templates within one system leads to a greater number of nucleation sites and active centres than do single-template systems; moreover, they influence the orientation, size, and shape of the final products. Therefore, SF combined with other proteins, e.g., COL [114,115] or polysaccharides, e.g., sodium alginate [173] and Chi [177,178,179] are able to better mimic the real mineralisation system than are single-protein systems. The use of natural spider dragline silks secreted by the daddy long-legs spider is of particular interest in terms of HA nucleation [180]. Spider dragline silk embodies excellent mechanical properties together with optimal AA sequences and a secondary structure based on the α-helix and the β-sheet.

A further innovative bone regeneration concept involves the use of magnetic nanoparticles concerning both diagnostic and therapeutic approaches. It has been demonstrated that iron oxides (Fe_2_O_3_, Fe_3_O_4_) enhance HA crystal radiopacity together with the proliferation activity of osteoblasts [181,182]. Inspired by this concept, Cojocaru et al. [183] prepared a composite based on a combination of biopolymers (Chi/HYA/BSA/GEL), CaP, and magnetic nanoparticles (Fe_3_O_4_). The proteins in the biocomposite, BSA and GEL, had specific tasks, i.e., the promotion of biomineralisation and the enhancement of cell adhesion, respectively. In addition, BSA exhibits defined AA sequencing and a molecular weight comparable to that of human serum albumin (HSA), while COL and GEL are difficult to define due to their diversity.

Salama and El-Sakhawy studied other creative protein/polysaccharide material combinations for the purposes of biomimetic CaP precipitation in the form of two-protein applications employing e.g., animal protein-wool/cellulose (W/Cel) [184] and plant protein-soy protein/cellulose (SP/Cel) [185]. Both W/Cel/HA and SP/Cel/CaP composites displayed good cytocompatibility as proved by in vitro studies using animal fibroblast baby hamster kidney cells. The utilisation of biodegradable plant proteins particularly offers significant potential in terms of tissue engineering applications and, moreover, the conversion of green biomass into useful products will have a positive impact on the economy without harming the environment. Zein, a major protein of corn, displays an amphiphilic character due to its unusual AA sequence, which contains highly hydrophobic residues, as a result of which it readily disperses in alcohol−water mixtures and is very stable in aqueous media. Zhang et al. [186] employed zein in their study on biomimetic mineralisation in the form of an insoluble protein monolayer on the air-liquid interface of 10 times-concentrated simulated body fluid. Zein films were covered with a continuous CaP layer with flake-like morphology particles consisting of a mixture of DCPD and HA. The modulus and hardness of the zein films increased to 11.6 and 0.32 GPa, respectively, via mineralisation accompanied by an increase in surface hydrophilicity (the water contact angles decreased from around 63° to 20°). Shahlori et al. [187], however, identified a hemispherical CaP morphology in the presence of zein.

The field of microbiology also provides inspiration for biomolecule-derived mineralisation [188]. Bacteria have been implicated as factors in biogeochemical cycles for the formation of minerals in aqueous sediments. Microorganisms are capable of segregating Ca from Mg and actively nucleate carbonate apatite by means of specific oligopeptides under pH < 8.5 and [Mg]:[Ca] ratio > 0.1 conditions, i.e., under the same conditions as those of the human body. Nanobacteria present in human blood are involved in pathological calcification [189,190]. Bacterial flagella represent further “bottom-up” self-assembling model protein systems that can be used as novel “smart” basic building blocks for the construction of composite biomaterials that structurally mimic the nanoscale level of various tissues. Flagella fibres are composed of flagellin, protein with several domains (α-helical and β-sheet) with specific functions [191]. Their outer diameter is 12–25 nm, the diameter of the inner channel is 2–3 nm, and they have lengths of 10–15 µm. Purified flagella *E. coli* fibres with an anionic aspartate-glutamate loop peptide with 18 carboxylate groups were employed by Kumara et al. [191] to initiate the formation of HA nanoparticles. Cervantes et al. [192] proved that the cellular wall of the Gram-positive *Bacillus thuringiensis* bacterium is able to segregate and incorporate various ions from physiological fluids and to induce the mineralisation process by means of membrane potential. It is possible to insert a foreign peptide into the variable region of flagellin via genetic engineering techniques, thus leading to the display of the foreign peptide on the surface of the flagella. Li et al. [193] displayed representative type I COL domains including part of the N-C-terminal, the N-C-zone around the hole zone, and an eight repeat unit Gly-Pro-Pro (GPP8) sequence, similar to the central sequence of type I COL, together with eight negatively charged Glu residues (E8) and two characteristic BSP sequences. Following incubation in a super-saturated precursor solution, the flagella that displayed E8 or GPP8 sequences were found to be coated with a layer of HA and to generate bone-like biomaterials. In addition, bacteriophages, that exhibit helically chiral surfaces with periodically and precisely aligned anionic Glu and Asp residues, have the potential for use in the fabrication of biomolecular-inorganic hybrid layered nanostructures [194]. Bacteriophages are filamentous and semiflexible viruses (~10–100 nm long and ~7 nm wide) that specifically infect bacteria without inducing obvious toxicity or immune responses in human beings.

##### Utilisation of Waste Biomaterials

The re-use of waste biomaterials offers a way in which to effectively prevent both their over-accumulation and the exhaustion of natural raw materials. A number of diverse methods, and methods aimed at their modification, have been developed over recent decades so as to obtain BAPs [195,196]. In addition to the direct isolation of BAPs from various sources and the preparation of apatites via the reaction of extracted Ca^2+^ precursors with PO_4_^3−^ ions, CaPs can be prepared by means of in situ synthesis with the aid of naturally derived protein biomolecules and via synthesis employing protein-based biomembranes (Section Natural Protein/CaP Systems).

Chrome shavings, a currently under-utilised chromium-containing waste product generated in the leather industry, which are usually disposed of in landfill sites have the potential to provide an alternative cheap source of COL that can be extracted in the form of a hydrolysate following the removal of the chromium. Banerjee et al. [197] proved that this material is amenable to the biomimetic growth of CaPs due to the availability of a large number of unmasked –COO^−^ groups. Nayar et al. [198] considered biomolecules from food waste such as vegetable and fruit peelings, leaves, and flowers, which contain various biomolecules (proteins, enzymes, vitamins, and saccharides) that enable the in-situ synthesis of nanosized CaP nanoparticles. Moreover, an eggshell extract has been found to facilitate a Ca/P precipitate molar ratio closest to that of living bone (1.67) due most probably to the natural calcium content of eggshells.

##### Synthesis of CaP Nanoparticles

The fact that polymers such as proteins are able to sequester ions, control nucleation and nanoparticle growth, and guide the self-assembly of nanoparticles renders them ideal for use for particle synthesis purposes. A template is needed to achieve the controlled morphogenesis of polycrystalline materials from amorphous precursors [199]; otherwise, in the absence of a template, the aggregation of nanocrystals leads to the formation of uncontrolled morphologies [200].

Besides the proteins, it is suggested that complexing effect of organic molecules, such as AAs and chelating reagents, with Ca^2+^, can modulate the size of CaPs. Steric structure of template is believed to play one of an important role in shape and size of CaP precipitates. Fibrous templates can lead to a more needle-like CaP, while globose templates, result in sheet-like CaP, as mentioned in Section 3.1.1. Compared with a linear structure, branched molecules and dendrimers can apparently decrease the crystal size, since CaP nucleation and crystallization may be localized in the exterior or interior of branched molecules. It is generally assumed that proteins regulate solution crystal growth via some sort of ‘epitaxial’ relationship between specific crystallographic faces and specific protein conformers. Amelogenin (AMEL) a protein for the control of the initiation, nucleation orientation, and growth of HA crystals in tooth enamel, can be mentioned such as an illustrative example. It was found that the strong interaction of AMEL with the (010) face of the OCP crystal, inhibits its growth in the *b*-axial

Direction, as it will be described in Section 3.3.1 in detail. Thus, generally, proteins may exhibit differing inhibiting or stimulating behaviours depending on materials and environment. Moreover, adjusting the initial pH of precursors was found to have a nonnegligible effect on the size and morphology of the final CaP products [68]. The influences of various factors such as chemical principle of protein, concentrations of Ca^2+^ and PO_4_^3^^−^ ions and proteins in solution, temperature, pH, and holding time on the morphology and crystallinity of the CaPs have been reviewed in detail by Lin et al. [199] or by Qi et al. [22]. It is more likely that final parameters of CaP precipitates are affected by the interplay of various factors and it is very difficult to distinguish between their significance.

**Figure 4:**
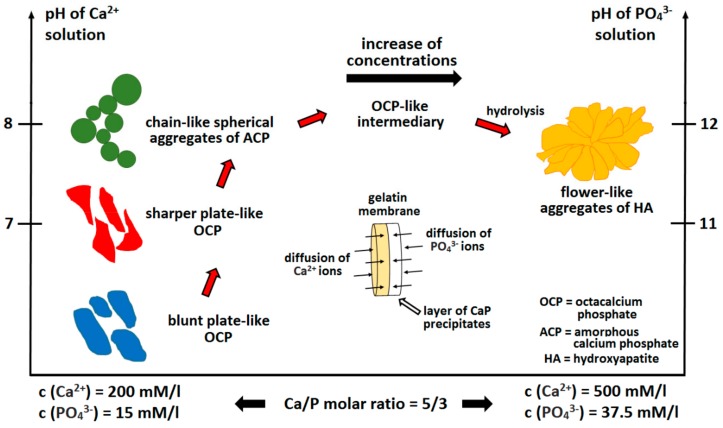
The formation of various CaP aggregates in GEL as a template by means of varying pH values and the concentrations of source ions, according to Teng et al. [201].

GEL has been used alone [201] and in combination with agar [202] for the preparation of various CaP aggregates (Figure 4) by means of varying both pH values and the concentrations of Ca^2+^ and PO_4_^3−^ ions [201]. CaP nanoparticles can be obtained via the removal of polymers by means of either calcination over 500 °C [201,203,204,205,206,207,208] or washing [202].

Han et al. [203,204] determined that with the increasing concentration of protein (BSA), there was no change in the composition of the products obtained and that the HA crystals adopted a mainly spherical form becoming more uniform and smaller with decreasing crystallinity. With the increasing temperature of calcination, rod-like HA crystals formed at around 600 °C. Zhao et al. [147,205] employed OVA as a natural biosurfactant in order to synthesise needle-like HA particles at room temperature. With further calcination up to 700 °C [205], particle agglomeration, together with a decrease in SSA, was seen to occur. The agglomeration ascribed to the removal of the OVA proved that proteins may serve not only as a template but also as a dispersant for preventing the agglomeration of crystal grains. Calcination allowed for the partial transformation of calcium-deficient HA to β-tricalcium phosphate (TCP) at a lower temperature (550 °C) as a result of the cooperative effect of the substitution of CO_3_^2−^ for PO_4_^3−^ ions, thus resulting in the nanoparticles adopting positive electrical charges.

In addition, complexing agents or surfactants are able to arrest crystal growth. The first successful attempt at the synthesis of CaP nanostructures using complexing agents based on Schiff bases (–RC=N–, naturally present in COL) was described by Mohandes et al. [209] who both suggested the growth mechanism and explained the formation of the various morphologies (nanoparticles, needle-like nanorods, and nanorods) with the assistance of various Schiff bases.

### 3.2. Coatings

Since various interactions at the interfaces of the implant/surrounding tissues always occur in surgical practice that employs implants, the surface properties of the implants constitute key parameters in terms of the biological response and further healing. Consequently, surface engineering techniques are applied to address surface modification aimed at ensuring the optimum surface biological and mechanical properties without altering the bulk properties. CaP coatings make up one of several options that are suitable for load-bearing applications. Introduced in 1976 [210], they can be used in various forms such as coatings, films, and layers in a large number of production techniques [211], and the approach to their use depends on the field of science and/or technology.

However, the mineral layers generated by most of the standard methods employed, such as plasma thermal spraying, are composed of large, partially molten CaP particles, which are prone to delamination as well as degradation in biological environments. High-temperature processes may also lead to the degradation of the underlying substrates subjected to coating and, thus, are not suitable for the coating of low melting point polymers, which are being increasingly employed in the field of tissue material engineering and for the covering of porous surfaces. In order to overcome these disadvantages, low-temperature coating methods can be applied [212] including a biomimetic coating technique involving the nucleation and growth of bone-like crystals via immersion in SBFs under physiological conditions (37 °C, pH = 7.3) [6]. CaP formation is governed by the surface characteristics of the materials and the immersion parameters, such as the composition of the SBF, ionic strength, pH, temperature, and immersion time. Since SBFs play a critical role in this process, the compositional aspects of various SBFs must be taken into account [213,214,215]. Biomimetic coatings can be applied to various types of materials including metals, ceramics, polymers, and organic/inorganic composite materials.

However, in some cases, the interaction between CaP molecules and matrices may result in poor physical bonding and, consequently, loosely attached nano-CaP particles may migrate into surrounding tissues, which, in turn, may provoke the activation of macrophages and result in damage to healthy tissue. In order to achieve successful CaP coating, the surfaces of the material need to be modified, e.g., via various organic compounds that chemically resemble non-collagenous glycoproteins and proteoglycans, which are able to serve as templates for CaP biomimetic precipitation.

#### 3.2.1. Metallic Substrates

Metallic substrates can be divided to bioinert (mainly titanium, steel, aluminium, and their alloys) and to biodegradable (e.g., magnesium and its alloys). Despite the favourable mechanical and chemical properties of most metals and their alloys, they do not form optimal biological or chemical bonds at the material-tissue interface.

Chakraborty et al. [216] compared a low-temperature biomimetic route with and without the use of globular protein—BSA on SS316 L stainless steel. They subsequently proved that the presence of BSA led to the adopted process mimicking the orientation and morphology of apatite crystals in natural bone. The surface without BSA exhibited poor coverage and discontinuity. While the physical adsorption of the organic template to the metallic surface appeared to be simple and flexible, generally this method results in the instability of the coating film [217]. Subsequently, Tapsir and Saidin [218] immobilised COL type I on a medical grade SS316 L surface grafted with a PDA film.

**Figure 5:**
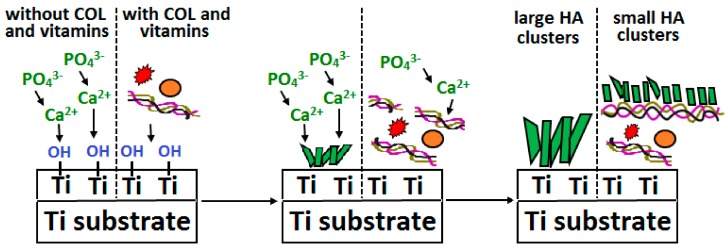
Comparison of the formation of various CaP aggregates and clusters on a Ti surface without and with modification via COL and vitamins as templates, according to Ciobanu and Ciobanu [222].

Titanium (Ti) and alloys thereof currently constitute the most commonly employed materials with respect to the manufacture of orthopaedic and dental implants and were among the first materials to receive attention in this respect by the scientific community. Since the mechanical properties of pure titanium are not sufficient for many applications, alloys with the general formula TiAlV must be employed, most frequently Ti6Al4V, which is available at 4 levels of purity depending on the iron and oxygen content. Titanium alloys are characterised by good mechanical properties, in particular a combination of high strength and low density. Since the Young’s modulus of these alloys is closer to that of bone than are austenitic steel and cobalt alloys, they are better able to reduce stress-shielding. Moreover, their excellent corrosion resistance is due to the simple formation of a naturally resistant passive titanium oxide layer (thickness ~4 nm), which is responsible not only for chemical stability, inertia, and corrosion resistance, but also for that fact that the osseointegration of Ti occurs without a negative tissue response [219]. Moreover, since it is known that titanium alloys are bioinert materials, the modification of their surfaces via the application of a material that mimics the bone extracellular matrix acts to enhance their bioactivity. COL suggests itself as a particularly appropriate material for this purpose prepared either in the form of an electrospun layer [220] or via immersion in a COL solution [221,222]. Sukhodub [221] proved that the application of COL on a Ti6Al4V substrate improved both the speed of reaction and crystallinity of the deposited layers. Ciobanu and Ciobanu [222] modified a supersaturated calcification solution containing COL via the addition of vitamins A and D3. Their results confirmed that the existence of these vitamins and COL in solution led to more rapid CaP precipitation on an alkaline pre-treated Ti surface. Moreover, they proved that COL together with the vitamins reduced the size of apatite crystals from ~500 nm to ~150 nm (Figure 5), thus resulting in enhanced apatite entity stabilisation on the Ti substrate. Tan et al. [223] immobilised GEL employing an MA-modified Ti surface via the photochemical approach, which resulted in stable surface coatings. Employing a combination of natural and synthetic polymers, the GEL/MA template was able to mediate the formation of an amorphous HA-like phase.

However, since COL and GEL derived from natural sources carries the risk of allergy, a recombinant COL-like protein has been applied with respect to Ti [224] and Ti6Al4V [225] substrate modifications by biomimetic CaP precipitation in SBFs. These studies [224,225] demonstrated that the modification of surfaces with short synthetic peptides resembling ECM proteins is capable of promoting the survival and differentiation of osteoprogenitor cells with varying degrees of potency. As well, peptide amphiphiles in the form of self-assembled lipopeptide nanofibres have been found to assert themselves so as to coat a Ti6Al4V foam [226].

Several studies [227,228,229], reported that the presence of albumin decreases the amount of CaP surface precipitation. However, conversely, other studies [183,216,230,231] proved the promoting effect of BSA on CaP precipitation. Moreover, a further study by Andesson et al. [232] employing fibrinogen proved the stimulating effect on CaP formation. Their results indicated that fibrinogen promotes CaP formation more readily than does albumin. The albumin molecule is smaller than that of fibrinogen, a fact which may be related to mineralisation processes. Areva et al. [233] confirmed that the formation of bonelike apatite may, in some cases, be hindered by the adsorption of proteins onto initially formed amorphous CaP centres, resulting in the subsequent alteration of the dissolution/reprecipitation processes required for the formation of poorly crystalline bone-like apatite. Proteins are able to adsorb selectively on different crystal facets of one crystal particle due to the presence of water molecules and surface hydroxyl groups.

Mineralisation on metal oxides and the approach to the control of the mineralisation process has been studied by Shou et al. [234]. They demonstrated the facet-specific mineralisation of nano-CaP: cover up {101} facets of anatase TiO_2_, while there were only a small number on {001} facets. It is thought that pre-adsorbed foetal bovine serum (FBS) protein on {001} facets may play a barrier role in preventing the sequential nucleation of nano-CaP. The discrepancies between the final conclusions of the various researchers may have been due to the use of different SBF compositions and operating procedures [40,216,235]. Zhao et al. [214] investigated the role of carbonates in carbonate-buffered SBF versus the SBF proposed by Kokubo [236] and the effect of BSA with different concentrations on the nucleation and growth of CaP from SBF. They determined that the presence of BSA strongly inhibited the formation of HA in traditional SBF, while HA could still be observed in carbonate-buffered SBFs. The inhibitory effect was found to be concentration dependent, with the complete inhibition of HA formation at 5 g/L of BSA.

It is not easy to explain the ambiguous results obtained concerning the behaviour of BSA in the CaP precipitation process. Recent studies conducted on COL/BSA systems [229,237] have determined that the CaP precipitation process involves a complex of possible specific mutual interactions between certain proteins in systems, and have proved that the isoelectric points of individual proteins together with the pH gradient play an important role in the CaP deposition process. D’Elia et al. [238] assessed the bioactivity of bone-like HA, TiO_2_, Ce-TiO_2_, and CeO_2_-TiO_2_ nanoparticles concerning the selective roles played by the presence of BSA with respect to the evolution of a biogenic apatite coating. They also studied the effect of material surface reactivity and the interfacial hydration thereof and demonstrated that these properties are responsible for bonding-site alteration and the surface charge density distribution, which, in turn, regulate the protein adsorption process. The specific BSA adhesion and the subsequent formation of a CaP coating are influenced primarily by the substrate identity. They concluded that BSA exerts two effects on the mineralisation of the HA substrate: (1) Active mineral surface sites efficiently coated with BSA slow down ionic diffusion to the crystal surface and (2) the BSA leads to the dissolution of calcium ions from the HA surface, thus reducing the co-precipitation of the new CaP layer.

Finally, it is important to consider surface physical characteristics such as topography, morphology, and roughness [228,232], all of which may exert a non-negligible effect on the nucleating behaviour of CaP and on the proteins and bacterials adhesion [239,240], cell proliferation, and differentiation [241].

A further variation concerning biomimetic precipitation together with the incorporation of functional molecules, electrochemical deposition, provides a potentially promising method due to a number of advantages, i.e., simple parameter controllability, short preparation time, simple equipment setup, and ease of operation. Zhuang et al. [237] demonstrated the controllable loading of BSA in mineralised COL coatings, the microstructures of which and the BSA loading quantity can be controlled by adjusting the parameters of the electrochemical process. Sun et al. [242] employed this procedure, which is used for the coating of OCP/COL composites, on an Ni/Ti shape memory alloy with high chemical stability and corrosion resistance.

Cells are directed towards attaching, differentiating, and spreading on the surface of implants via cell-binding or integrin-binding proteins such as bone sialoprotein (BSP) [243], which exhibits a high proportion of negatively charged residues that are able to interact with Ca^2+^ ions. Similarly, peptides are also able to interact with non-biological inorganic materials. Sano et al. [244] suggested that AA sequences such as RKLPDA (TBP) are able to interact electrostatically with the amphoteric TiO_2_ layer that forms on the surface of pure Ti; the interactions occurred between both positively charged Arg and Ti-O^−^ and negatively charged Asp and Ti-OH_2_^+^. Inspired by this suggestion, Kelly et al. [245] proposed a novel technology that utilised short peptides consisting of a combination of an HA binding peptide (E8) and TBP. Octaglutamic acid (E8) originates from sialoprotein through the extraction of negatively charged functional peptide sequences. Polyglutamic acid (PGlu) is able to provide multiple negative charges for binding to Ca^2+^ ions.

Magnesium (Mg) and its alloys [246] attract most of the recent attention in research aiming to develop temporary biomedical devices due to their acceptable biocompatibility. Mg alloys offer a low modulus of elasticity, close to that of the human bone but their relatively low strength compared to the conventional biocompatible metallic alloys limits the use of these alloys to low load-bearing applications. However, these alloys undergo a considerably fast degradation process in a physiological environment because the interface between the Mg-based implants and biological environment is very dynamic and this corrosion process could limit their biomedical applications. Mg dissolution results in the emission of hydrogen and hydroxyl groups that increase pH value. The reduction of corrosion rate can be diminished by post-processes such as heat treatment, mechanical/chemical modification, and coating deposition of Mg substrates [247]. Lin et al. [248] has employed PDA to decrease the corrosion rate of Mg and its alloys. The potentiodynamic polarisation and hydrogen evolution tests applied in their study demonstrated that the corrosion resistance of a modified AZ31 Mg alloy both significantly increased and promoted the proliferation of L-929 cells. Cui et al. [249] designed a biomimetic coating on the Mg alloy (AZ31B) based on the highly acidic dentin sialophosphoprotein (DSPP). Phosphophoryn (PP), a cleavage product of DSPP, performs an important role in various cellular activities. Moreover, PP is an acidic Asp-Ser-rich protein and is able to control the formation of CaP.

#### 3.2.2. Polymer and Ceramic Substrates

The biomimetic approach has also been employed for the coating of polymer composites and scaffolds. Most synthetic polymers, e.g., polyesters, are unable to interact specifically with cells due to their relatively high hydrophobicity and lack of functional groups for the attachment of biologically active molecules, concerning which surface modifications via the application of various hydrophilic coatings may provide a reliable solution. One such coating, layer-by-layer (LBL) deposition, provides a polyelectrolyte multilayer surface coating via the alternating adsorption of positively and negatively charged species from aqueous solutions, especially with concern to substrates with irregular shapes and inner structures. Moreover, this method allows for the tailoring of a wide variety of differing biofunctional properties. Abdelkebir et al. [250] reported that LBL films fabricated from anionic ChS and cationic poly(L-lysine) (PLL), with different terminal layers, provide effective templates for the control of the deposition kinetics and structures of CaP coatings. Zhao et al. [251,252] developed a unique multilayer system made of LBL based on COL type I with intrinsic cross-linking capacity via the use of oxidised ChS as a polyanion. They proved that this LBL film could be potentially employed as a tool for the promotion of the biomimetic mineralisation of CaP. Consequently, a further study [253] by the same team involved the deposition of CaP on poly-(lactide-co-glycolide acid) scaffolds by means of the prior coating of ChS/COL multilayers on the scaffold in the form of a nucleation material. The resulting fabricated composite exhibited the ability to promote the osteogenic differentiation of bone mesenchymal stem cells through the up-regulation of osteogenic marker genes. Li et al. [254] also applied the LBL technique for the fabrication of fibrous PCL mats, followed by surface modification with GEL; the deposition of CaP consisted of a mixture of DCPD and apatite. The proliferation rate of preosteoblastic MC3T3-E1 cells on the modified PCL scaffold was found to be 1.9 times the proliferation rate on the non-modified scaffold. The same material combination was employed by Tarik Arafat et al. [255] who fabricated PCL/TCP via the screw extrusion system followed by coating with carbonated HA/GEL. Employing porcine bone marrow stromal cells (BMSCs), they proved that such a material combination stimulated the osteogenic differentiation of BMSCs to a greater extent than did PCL/TCP and CHA-coated PCL/TCP scaffolds.

Polystyrene (PS), one of a number of commonly used polymers, is bio- and blood-compatible and is used widely for the manufacture of cell culture plates since it can easily be moulded into various shapes and exhibits excellent transparency and physical properties. Moreover, it has recently been determined that PS also lends itself to computer-aided design and 3D printing approaches. The coating of PS surfaces with CaP has the potential to significantly expand their application with respect to various biomedical procedures. However, PS does not display effective functional groups for the purpose of biomimetic CaP mineralisation. Iijima and Hashizume [256,257,258,259] developed methods for the functionalisation of PS substrates with CaP. In order to investigate the versatility of this method, they investigated the effect of the adsorption of various proteins such as HSA [257,258,259,260], human immunoglobulin G (hIgG) [256,257], COL type I, hen egg white lysozyme, and PGlu [259]. They also studied the influence of protein/CaP coatings on the adhesion and growth of hMSCs [259,260].

Chen et al. [261] modified the surface of poly(L-lactic acid) (PLLA)/nβ-TCP composites by means of a biomimetic GEL/HAP coating, which was found to substantially improve MC3T3-E1 cell adhesion, proliferation, and osteogenic differentiation; moreover, in vivo tests using a rabbit model demonstrated an improvement in osteointegration and the significant promotion of bone regeneration.

Despite the favourable mechanical and chemical properties of alumina (α-Al_2_O_3_), it forms no biological or chemical bonds at the material-tissue interface. Chakraborty et al. [235] compared a low-temperature biomimetic route with and without the use of a globular protein—OVA on the α-Al_2_O_3_. They proved that the presence of OVA resulted in the increased regularity of crystal plates and the formation of carbonate-containing HA. Schickle et al. [230] proved the positive influence of BSA on HA precipitation on zirconia (commonly used in the dental industry—it possesses excellent biological, mechanical, and aesthetic properties but is practically inert).

### 3.3. Reconstruction and Regeneration Strategies with Concern to Dental Applications

Dental caries constitute a major healthcare problem, with respect to which nanotechnologies enable a number of innovative preventative, reversal, and restorative dental applications [262], some of which exhibit an antimicrobial effect that helps in the preventive stage and others the potential for remineralisation, thus inhibiting early lesion progress. The development of dental caries is a multifactorial dynamic process caused by an imbalance between demineralisation and remineralisation [263,264]. If this delicate balance is upset, white spot lesions (the loss of enamel transparency) may appear, which proceed into the underlying dental structures—dentin and dental pulp [265].

Enamel is acellular and consists of more than 95–97% of highly organised HA crystallites and less than 1–2% of organic material and water, thus making it the hardest tissue in the human body. The basic microstructure of enamel contains nanosized fibril-like carbonate HA crystals that are tightly packed together to form prisms. These unique prism structures run approximately perpendicular from the enamel-dentin junction towards the surface of the tooth. Dentin, however, which resembles bone, is composed of 70% HA minerals, 20% of an organic matrix (mainly COL), and 10% of water and is permeated by dentinal tubules, which radiate from the pulp cavity towards the enamel–dentin junction. The tubules are surrounded by dense peritubular dentin composed of mineralised COL fibrils [266]. The biomineralisation of enamel and dentin is a highly regulated process involving protein–protein, protein–mineral, and cell membrane interactions; moreover, it requires precise genetic control [267]. The increasing demand for aesthetic restorative treatment has led to the development of adhesive integrated materials (such as adhesive systems and composites) and techniques aimed at restoring the natural appearance of teeth, especially in the anterior segment [268]. “Smart” adhesive materials, that are required to interact therapeutically with dental hard tissues, reduce degradation together with remineralisation ability [269]. During the last several years, different biomimetic systems have been developed for enamel and dentin remineralisation based on amelogenin, other peptides, dendrimers, amino acids, calcium phosphate nanoparticles, and others [270,271]. This approach is known as “filling without drilling” [272].

#### 3.3.1. Systems Based on Amelogenin

**Figure 6:**
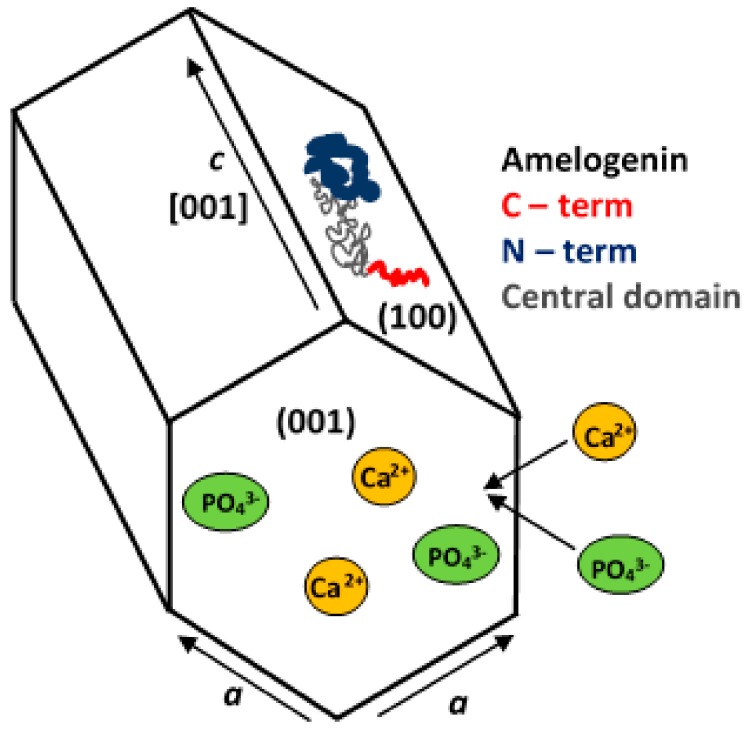
Model of the amelogenin (AMEL)-controlled growth of hydroxyapatite (HA) crystals.

Amelogenin (AMEL) is the main secretory product of ameloblasts in the extracellular space between ameloblasts and dentine for the control of the initiation, nucleation orientation, and growth of HA crystals [273,274]. It makes up more than 90% of the organic component in enamel and is composed of N-terminal tyrosine (Tyr) and histidine (His)-rich domains, a large central hydrophobic domain, rich in Pro, and a charged hydrophilic C-terminal (−Thr-Lys-Arg-Glu-Glu-Val-Asp) “tails”. AMEL is able to self-assemble to form oblate nanoparticles comprised of approximately 100 monomers so as to play a direct role in the initiation of nucleation and crystallisation via the transitional formation of amorphous CaP-AMEL nanospherical complex clusters, which co-assemble as chains of nanoparticles that then evolve into long co-aligned stable densely packed HA crystals. Martinez-Avila et al. [275] determined that individual monomers of AMEL are able to self-assemble into dimers and, further, into building blocks in a pH range of 4–6 due to the formation of ion bridges through protonated His residues. It is suspected that AMEL C-terminus interactions dominate the bonds to the Ca^2+^ ions of HA. Friddle et al. [276] proved that binding to the (100) face is strongly preferred to binding to (001). Thus, AMEL-assisted HA formation leads to elongation along (001) via the selective inhibition of (100) growth; i.e., by inducing elongation in the [001] direction (Figure 6). It has also been proved that the addition of AMEL results in a remarkable increase in the size and aspect ratio of OCP due to the strong interaction of AMEL with the (010) face of the OCP crystal, which inhibits its growth in the *b*-axial direction (width) [277,278].

The robust interface between natural and synthetic enamel crystal promotes strong bonding between the new-grown layer and the original tooth interface [279]. A number of studies have investigated a novel AMEL-based peptide composed only of the functionality AA residues of the N-terminus and the hydrophilic C-terminus [280,281,282,283]. Dogan et al. [284] demonstrated that the presence of 22-amino acids segment of AMEL-derived peptides (ADP5) facilitates delivery to the surface of the tooth and the incorporation of F^-^ ions into the remineralised layer even at low fluoride concentrations. Uskoković et al. [282] established the physicochemical and biochemical conditions for the growth of apatite crystals under the control of a recombinant AMEL matrix (rH174) and proved that, despite their mainly hydrophobic character, AMEL nanospheres acted as a nucleating agent for the crystallisation of CaP. Moreover, it has been proved that AMEL-based synthetic peptides are able to enhance the remineralisation of germinal enamel lesions in a dose-dependent fashion [273].

#### 3.3.2. Systems Based on Amino Acids and Peptides

It is believed that AAs such as Glu and Gly may interact with HA over a thin ACP layer [285,286,287,288,289] and that they are able to guide HA/ACP core-shell nanoparticles into oriented and ordered arrays. AMEL contains 15–20% Glu in the AA composition. The biomimetic construction of enamel-like apatite material is attained via the cooperation of nanoparticles and Glu on an enamel substrate in vitro under physiological conditions. Li et al. [290] proved that the artificial apatite layer shares exactly the same crystallographic and tectonic properties as natural enamel.

Zhou et al. [291] applied a polydopamine (PDA) coating (derived from phenylalanine) to demineralised enamel and dentin surfaces in order to evaluate the effect on dental remineralisation. No significant difference was determined in terms of the remineralisation of enamel in the presence of PDA. However, a significant difference was evident with respect to dentin remineralisation following the application of PDA. In the case of enamel, the substrate consisted of an inorganic environment upon which precipitation crystals nucleated directly and grew on pre-existing enamel HA crystals. With concern to dentin, however, the remineralisation substrate consisted of COL, the remineralisation of which is an organic-mediated process with heterogeneous crystal nucleations and with PDA representing new nucleation sites.

Silk sericine (SS) provides a further natural source that allows for the assembly of HA with an enamel prism-like structure. SS forms the outer part of silk and contributes around 20–30% of the total weight of the cocoon and, moreover, it is characterised by a high content of Ser and 18 AAs, including essential AAs. The original spherical shape and the subsequent conformational transition of SS from a random coil to a β-sheet provide the ability to modulate the enamel-like HA structure. The homogeneity of the assemblies and the particle size, morphology, and crystallinity may be influenced by the SS concentration and mineralisation time [292], the solution pH values, and the temperature [293].

Bioactive agents based on milk products have also been developed aimed at enhancing the remineralisation of enamel [294] and dentine [295,296]. A multifactorial anticariogenic agent based on a nano-complex of the milk protein casein-phosphopeptide (CPP) with ACP, is even available commercially in the form of a paste (Tooth Mousse, GC International, Itabashi-ku, Tokyo, Japan).

Owing to difficulties with the extraction and purification of natural proteins, research is increasingly being directed towards protein analogues. Proteins and peptides with self-assembly ability [297,298] in particular are able to create various space structures and can be used inter alia in regenerative applications. Various genetically engineered peptides for inorganics, known as GEPIs, are being isolated and designed at the molecular level by means of phage display methodology so as to provide evolutionary and rationalised peptide sequences for HA-binding purposes [299,300,301,302].

Although the mechanism is not yet fully understood, DMP1, the essential acidic NCP in dentin and bone, which contains a large number of Ser (22%), Glu (15%), and Asp (13%) amino acids, is believed to play an important role in the mineralisation of such tissues. It has also been used in the recombinant form [303,304]. While recombinant DMP1 exists as a random coil, following the binding of the calcium, the C-terminal domain adopts a β-sheet secondary structure and gradually self-assembles into oligomers and microfibrils. Beniash et al. [304] determined that, interestingly, with respect to DMP1-rich peritubular dentin, which lacks COL fibrils, the HA crystals are organised into bundles with their *c*-axes co-aligned with the axis of the bundle. DMP1 is able to control mineral organisation outside of the COL fibrils.

Synthetic recombinant proteins are based on various repetitive peptide sequences. Peptides with sequences of Asp-Ser-Ser (DSS), based on the dentin phosphoprotein (DPP) sequence, exhibit a high affinity to CaPs and they can be prepared in the form of multiple-modifications, e.g., 3DSS [305] and 8DSS [306]. Other modifications are related to changes in the type of AA, e.g., the triplet repeating Asparagine (Asn)-Ser-Ser peptide (3NSS); the COOH group in the Asp is subsequently replaced by the CONH_2_ group in the Asn [307,308]. A further candidate consists of the self-assembling P11-4 peptide [309,310,311,312,313] that consists of natural AAs such as glutamine (Gln), Glu, Phe, Trp, and Arg. P11-4 is able to diffuse into lesions, self-assemble spontaneously, and produce 3D gels comprised of β-sheet aggregates, thus enhancing the attachment of Ca^2+^ and PO_4_^3−^ from saliva. Conversely, another study, that compared the effect of a self-assembling peptide on an artificial enamel caries lesion with fluoride, and caries infiltration, determined that the self-assembling peptide was not capable of either inhibiting further lesion progression or masking the lesions [313]. Self-assembling peptide amphiphiles (PAs) possess significant potential as nanofabrication templates for e.g., biomineralisation, as mentioned in Section Synthetic Peptide/CaP Systems. Moreover, PAs that display an integrin-specific RGD moiety (also mentioned in Section Synthetic Peptide/CaP Systems) have been shown to promote cell adhesion, proliferation, and differentiation. The density of RGD epitopes can be controlled via the application of a form of molecular architecture (branched, linear, cyclic). With respect to enamel regeneration, Huang et al. [314] employed a branched PA-bearing RGD to provide a synthetic extracellular environment similar to that prevailing at the time of ameloblast differentiation. In addition, the branched architecture of PAs was found to demonstrate greater signalling capacity than its linear counterpart.

#### 3.3.3. Systems Based on Dendrimers

Dendrimers consist of highly branched polymers and possess internal cavities, a large number of reactive end groups, and a well-defined size and shape, and take the form of repeating, symmetrically branching units (monomers) bound radially to the core, which may be a small molecule or a linear polymer and which exhibits multiple binding sites. Individual monomers attach to these binding sites allowing for the formation of so-called dendrimer generations. A higher generation is formed with each additional layer of monomers.

Poly(amidoamine) (PAMAM) dendrimers, known as artificial proteins, constitute one of the most well-known forms of dendrimers and are considered to be NCP analogues due to their mono-dispersed molecular weight that lies within the COL size retention range, and their well-defined steric structure. The PAMAM core consists of a diamine (commonly an ethylenediamine), which reacts with methyl acrylate and another ethylenediamine so as to form generation-0 (G-0) PAMAM. Further reactions create higher generations, which tend to exhibit different properties. Lower generations can be considered to be flexible molecules with no appreciable inner regions, while medium-sized generations (G-3 or G-4) feature an internal space that is essentially separated from the outer shell of the dendrimer.

It has been proved [315,316,317,318] that the precise mechanism of HA crystallisation can be attributed to the localisation of the nucleation site: External or interior PAMAM. Various crystal morphologies can be prepared via the adsorption of different dendrimers onto the specific faces of growing crystals, which alters the relative growth rates of the different crystallographic faces and results in differing crystal habits. Chen et al. [319,320] demonstrated that PAMAM dendrimers capped with carboxylic acid can be adsorbed onto enamel crystals; they were subsequently used to remineralise the surface of etched enamel in vitro [321,322] and for the bioinspired mineralisation of human dentine both in vitro and in vivo [323]. Fourth-generation PANAM (G4-COOH), in particular, provides a general strategy for the preparation of various promising restorative materials for biomineralised hard tissues such as bone and teeth. In addition, Yang et al. [324] reported an amphiphilic PAMAM dendrimer that performed a function similar to that of AMEL in the oriented growth of HA crystals in vitro.

#### 3.3.4. Other Systems

Other systems based on various organic additives and specialised techniques have been developed for the remineralisation of enamel and dentin.

Dentin adhesives rely on the micromechanical entanglement of resin polymers within the partially or completely demineralised COL in the dentin for the retention of resin composite fillings. This infiltration process creates a so-called interdiffusion zone. Polyacrylic acid (PAA), analogous to NCPs, reproduces the structural hierarchy of the ordered apatite deposition in the COL matrix [325,326]. Polyvinylphosphonic acid (PVPA) consists of a polyanion that mimics phosphoproteins such as DMP-1, PP, and BSP. These acidic resin monomers with carboxylic or phosphoric acid functional groups constitute so-called self-etching adhesives, the use of which leads to the production of zones of partially demineralised dentin containing seed apatite crystallites. Kim et al. [327] discovered that in the case of remineralisation media with no biomimetic analogues, remineralisation was observed only in partially demineralised COL matrices, most probably by means of epitaxial growth via top-down crystallisation. Conversely, the intrafibrillar remineralisation of completely demineralised COL matrices via bottom-up crystallisation was identified with the presence of biomimetic analogues in the remineralisation medium. While the top-down approach involved epitaxial growth over the seed crystallites, the bottom-up approach utilised matrix protein biomimetic analogues to stabilise the ACP nanoprecursors and template apatite nucleation and growth within the COL matrix [328]. Abuna et al. [329] examined the bonding performance and dentin remineralisation potential of an experimental adhesive containing CaP micro-fillers and self-etching primers doped with phosphoprotein biomimetic analogues and/or sodium trimetaphosphate. The biomimetic mineralisation of caries-like lesions has recently been reported employing Portland cement in the presence of PAA and polyvinylphosphonic (PVPA) acid-containing simulated body fluid (SBF) [330] or PAA and sodium tripolyphosphate-containing SBF [331]. Tay and Pashley studied the biomimetic remineralisation of acid-etched dentin disks by means of applying PAA and PVPA acids to a Portland cement-phosphate-containing fluid system in order to regulate apatite nucleation and growth [327,330]. Wu et al. [332] investigated the characteristics of PAA adsorption to desorption from COL type I and tested the mineralisation ability of PAA-bound COL. Busch [333] performed in vitro experiments that revealed that the diffusion of suitable ions through a GEL layer onto a tooth surface induces the mineralisation of FA layers with a structure similar to tooth enamel. The application of glutaraldehyde (GA) represents an unusual approach to inducing the biomimetic remineralisation process for the reconstruction of the mechanical properties and biostability of demineralised dentin. GA is widely used for cross-linking purposes in COL chemistry. However, some studies have shown that aldehyde-treated tissue may be subject to calcification [334,335]; while this form of mineralisation is undesirable with respect to soft-tissue implants, it may be advantageous in terms of the remineralisation of dentin. It has been proved that concentrations of GA of ~5% enhanced both the stability of the resin-dentin interface and bond durability [336,337,338]. Chen et al. [339] investigated biomimetic remineralisation via the application of GA in order to reconstruct the mechanical properties and biostability of demineralised CO, and proved that this procedure is able to promote dentin biomimetic remineralisation, resulting in improved mechanical properties and biostability. Despite the extensive research performed to date however, serious challenges remain with respect to the application of biomimetic strategies in the fields of dentistry and the material sciences.

### 3.4. Smart Devices

With respect to biomaterials, the term “smart” concerns the nature of the interactions between biomaterials and surrounding tissues and cells. Since such interactions include stimulating and inhibiting effects on cells and tissues over relatively prolonged periods, certain material modifications are applied to 3D scaffolds/composites and the preparation of drug delivery systems [16] in order to regulate the bulk and surface properties. Such material arrangements involve the adjustment of the bulk and surface properties of biomaterials so that they mimic the chemical and physical properties of the ECM together with the organisation thereof at the nano- or micro-scale. ECM molecule surface modification employing e.g., full sequence recombinant proteins or engineered peptides, is capable of enabling multiple interactions and events. Delivery biomaterials have been developed in various forms including porous foam scaffolds, hydrogels, nano/microfibres, and nano/microparticulates and combinations thereof in order to enable the loading and delivery of biofactors. The delivery strategy is determined “smartly” depending on the type of molecules to be delivered and can be adjusted so as to be released sustainably or to provide the sequential delivery of multiple factors.

The last decade or so has witnessed a significant increase in the level of interest expressed by researchers in organic/inorganic constructs with specific functions. Such “smart” constructs are able to assume the role of nanoreactors, i.e., one of the simplest forms of cell mimicking, that contain cavities in which chemical reactions are able to take place. While the construction of artificial cells remains a fantasy, more simple systems involving micelles, vesicles, and other molecular assemblies with specific functions may be capable of serving as devices with respect to a number of applications.

#### 3.4.1. Delivery Systems

Delivery systems can be designed via the introduction of external therapeutic molecules (drugs, proteins, and genes) into a structure in a way that allows for the sustainable and controllable delivery (and even time-dependent and sequential delivery) of multiple biofactors [16,340]. Therapeutic activity is attained either in direct contact with cells through cell membrane receptors, following cellular uptake or even following penetration into the nucleus. Those biofactors incorporated into an internal structure during the preparation of biomaterials are more suitable in terms of attaining long-term therapeutic effects in a more sustainable and time-dependent manner. Otherwise, biofactors that bond or are adsorbed on pre-formed biomaterial surfaces are aimed towards direct contact with cells. The biointerface constitutes the key location at which most of the initial events occur between the biomaterial and the host molecules. Such processes occur via the dissociation of surface molecules/ions, protein interactions, and cell anchorage and adhesion. The fact that biointerface control via ECMs makes up one of the most effective ways in which to provide scaffolds and implants with smart cell instructive functions has led to a significant increase in the intensity of the study of ECM-mimicking proteins over the last decade or so [341,342].

Biomimetic scaffolds and coatings are able to serve both as biocompatible elements and carrier and delivery systems. However, although such systems exhibit good osteoconductive properties, they do not directly confer a sufficient degree of osteoinductivity. In order to overcome this drawback, therefore, new methodologies are being developed aimed at improving the degree of calcification and enhancing both the osteoinductive effect and bone regeneration via the incorporation of drugs or biologically active molecules such as proteins and genes into the mineral coatings [340]. Hence, the introduction of the application of osteogenic growth factors such as BMP-2. However, since many materials exhibit only short-term burst release, a carrier coating that is able to release BMP-2 sequentially could provide a potential solution to the sustained release issue. If low doses (~400 ng/mL) of BMP-2 were successfully incorporated into the CaP during the co-deposition process, the prepared coatings would be able to deliver the protein in a sustainable manner for up to 20 days [343], thus enhancing the degree of osteoinductivity [344]. In order to further enhance osteoinductivity, Zheng et al. [345] developed a novel “osteoinducer” by biomimetically assembling a CaP layer-by-layer and co-precipitating BMP-2 into CaP granules, via which they proved the enhancement of ectopic bone formation in a rat model. A later study by the same team [346] proved that a BMP-2/CaP system is able to serve as an independent osteoinducer when mixed with osteoconductive biomaterials based on CaPs. BMP-2 was incorporated into the outermost layer of an inorganic BMP-2/CaP crystalline lattice, whereupon very low doses of BMP-2 were released slowly and steadily as the BMP-2 particles underwent degradation at the site of implantation. Subsequently, the same team [347] proved that BMP-2/CaP enhanced the osteoinductivity of deproteinised bovine bone (DBB) during healing in a sheep model. Many natural organic templates can be used for the delivery of BMP-2 such as silk fibroin [348], fibrin [349], COL and GEL [350,351,352,353,354], and so on.

**Figure 7:**
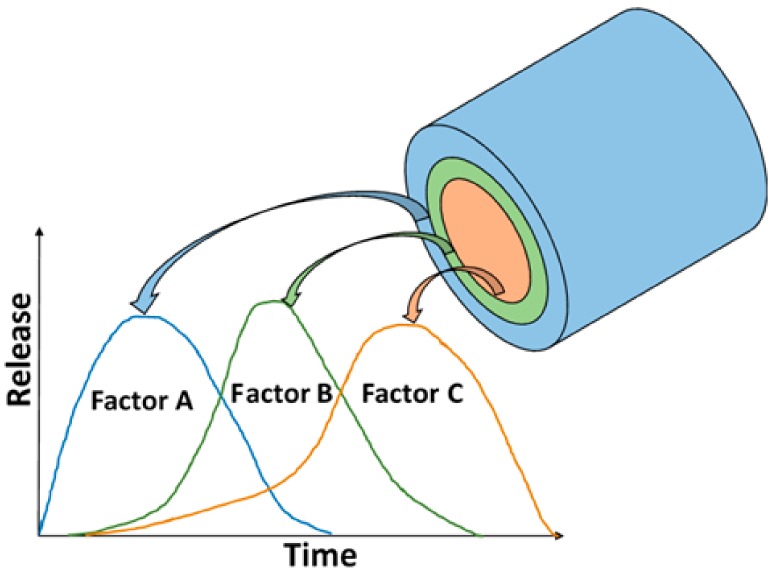
Schematic illustration of layered composite biomaterials with the “smart” sequential delivery of multiple factors.

A delivery coating for use in synthetic bone grafts has been developed to provide for the sequential delivery of multiple osteoinductive factors so as to better mimic certain aspects of the natural regenerative process. Fibroblast growth factor-2 (FGF-2) and bone morphogenetic protein-2 (BMP-2) constitute the two molecules that are best able to deliver sequentially to cells due to the differing biological activities of these two factors [355]. However, while they exert synergistic effects when used together, such co-delivery materials are not necessarily always successful [356]. Although BMP-2 is anabolic for bone formation, high doses may result in inflammation, ectopic bone formation, osteolysis, seroma formation, and an increase in the risk of malignancy; thus, the need remains to increase the efficacy of lower doses of BMP-2. Gronowicz et al. [357] developed a sequential delivery system for FGF-2 and BMP-2 based on the principal illustrated in Figure 7. The BMP-2 was applied prior to the coating process directly upon a synthetic bone graft and, subsequently, employing the LBL technique, a CaP-polyelectrolyte multilayer (PEM) composed of PLL/PGlu was deposited followed by the adsorption of fibroblast growth FGF-2 into the PEM. The addition of the CaP layer to the PEM delayed access to the BMP-2 and allowed FGF-2-stimulated progenitors to populate the scaffold before differentiating in response to BMP-2, thus leading to the enhanced healing of the bone defect. The involvement of cells in the degradation process makes up a further important aspect in standard release studies involving elution into physiological solutions, concerning which Alhamdi et al. [358] investigated how the various parameters of a CaP/PEM system such as the number of PEM layers, the structure of the PEM molecules, and the thickness of the CaP influence the kinetics of cell access to the factors embedded in the CaP/PEM coating.

A further recently developed concept is based on a combination of classical BMP-2 with natural biomolecules such as Icariin [359], a typical flavonol glycoside and a strong anabolic agent, which enhances osteogenic differentiation while inhibiting osteoclastic formation. It is proposed that this combination will promote bone regeneration when loaded into a CaP cement or ceramic material.

Antibiotics constitute one of the most commonly applied medical treatments of our times. While many systems based on COL/CaPs as the drug delivery mechanism have been designed, the most popular system consists of the mixing of individual components [360,361,362]. Pon-On et al. [363] prepared such a system via the biomimetic precipitation of calf COL with vancomycin (V) and Ca^2+^ and PO_4_^3−^ ions employing ultrasonication. The loading efficiency for the V was around 78% and the period of sustained release was over two weeks.

A number of breakthroughs have been made with respect to cancer immunotherapy over the past decade or so. The synthesisation of selenium oxyanion-substituted hydroxyapatite (SeHA) provides a promising approach to the treatment of bone cancers through its reducing the probability of recurrence as a result of the significant effect exerted by selenium on the induction of cancer cell apoptosis. It has been proved that SeHA materials might, therefore, serve as an implant material constituent that provides both a scaffold for newly grown bone tissue and inhibits the growth and proliferation of tumour cells. Wang et al. [364] fabricated an SF/SeHA scaffold and proved that biomimetic coprecipitation provided a successful approach to the preparation of biocomposites that presented a high level of cell proliferation activity and the slow and stable release of the selenite. The release profile was strongly influenced by the SF that adhered to the surface of the HA.

The application of checkpoint inhibitors is currently gaining in popularity and, recently, they have been proved to work synergistically with therapeutic anti-cancer vaccines in the training of T cells to fight directly against tumour cells endogenously. Biomimetic nanotechnology offers the potential to enhance anticancer immunity via the co-delivery of immunogenic antigen materials and adjuvants to antigen-presenting cells. Necroptotic tumour cells are capable of releasing danger-associated molecule patterns such as heat shock proteins due to their being more immunogenic than native tumour cells. Inspired by this strategy, Kang et al. [365] designed and prepared a nano-sized “artificial necroptotic cancer cell” via biomimetic coprecipitation that comprised a phospholipid bilayer and a CaP core as a flexible vaccine platform for the co-delivery of cancer membrane proteins, signal augmenting elements, functional peptides, and adjuvants based on oligodeoxynucleotides. Photodynamic therapy is a form of phototherapy involving light and a photosensitising chemical substance and is employed in conjunction with molecular oxygen to elicit cell death. It is also able to treat cancer cells. CaP-reinforced photosensitiser-loaded polymer nanoparticles have been developed for photodynamic therapy purposes by Lee et al. [366]. Chlorin e6 (Ce6)-loaded core–shell–corona polymer micelles of poly(ethylene glycol)-b-PAsp-b-poly(L-phenylalanine) were employed as the CaP mineralisation template. The CaP mineral layer effectively inhibited the release of Ce6 from the Ce6-loaded mineralised nanoparticles at a physiological pH value. At a pH value of 5.0, the release of Ce6 was enhanced owing to the rapid dissolution of the CaP, and the cell viability of breast tumour cells was found to decrease dramatically with increasing irradiation times.

**Figure 8:**
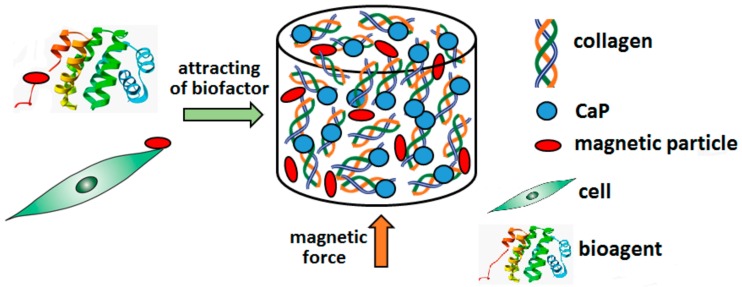
Schematic illustration of magnetic COL/CaP scaffold according to Bock et al. [367].

Controlled delivery remains a serious challenge with respect to the use of conventional scaffolds in the field of tissue engineering. Bock et al. [367] fabricated a conceptually new type of bone graft substitute based on COL/CaP, i.e., a magnetic scaffold (Figure 8) prepared via a dip-coating biomimetic precipitation process, which is able to attract and extract various bioagents in vivo via a driving magnetic force. This novel concept involves the use of magnetic iron oxide nanoparticles, which are functionalised with bioagents so that they can be attracted by the magnetic scaffold (which might be considered to be a fixed “station” that can be reloaded repeatedly following implantation). Magnetite (Fe_3_O_4_) is the most commonly employed magnetic material with concern to biomedical applications due to its superparamagnetic behaviour at room temperature.

#### 3.4.2. Smart Constructs

As mentioned in Section Natural Protein/CaP Systems, bacteria and bacteriophages are able to successfully serve both as biomineralisation templates and for use in the fabrication of biomolecular–inorganic hybrid nanostructures. Microorganisms exhibit low generation times, simple growth conditions, the potential for mass multiplication and scalability, and inherent resistance properties against various environmental conditions. It is thought that microbial biotemplates may, in the future, be employed in new concepts such as non-toxic, high-conductivity, organic-based electronic equipment such as transistors and supercapacitors. Moreover, the atomic layer deposition of various oxides on bacterial pili has been reported with respect to potential application in biosensors as functional electrodes [303]. To the best of the author’s knowledge, the literature has not yet described such applications for use in systems based on bacteria/HA; however, it is only a question of time before such systems are used in the production of functional nanoelectronic materials.

Since doped or substituted HAs are also used as fluorescent materials, it is thought that HA-based photoluminescent (PL) materials might provide an ideal bioimaging agent. Moreover, already-developed PL bioceramics provide huge potential with respect to the fields of biosensing and biooptoelectronics. A study by Chung et al. [368] synthesised a COL/HA nano hybrid that emitted a sustained PL light in contrast to pure COL, concerning which bright fluorescence was rapidly quenched.

**Figure 9:**
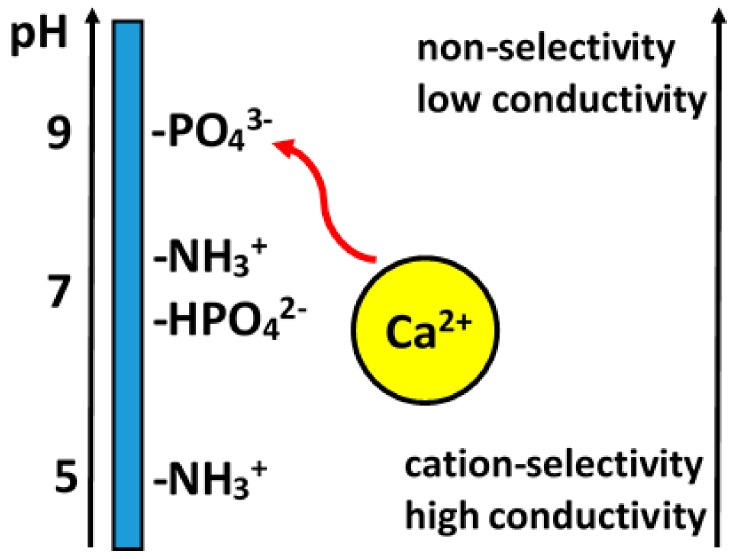
Schematic illustration of PET membrane modifying by O-Phospho-l-Tyr (OPLT) with functional groups according to Xu et al. [369]. System shows “smart” selective ion transport depending on pH value.

Calcium-responsive ion channels play crucial roles in a large number of biological events. Xu et al. [369] introduced a new idea concerning a multiple signal-induced ion gating for use in nanochannel device applications (Figure 9). They designed and fabricated a biomimetic artificial smart-responsive ion nanochannel system based on intelligent O-Phospho-l-Tyr (OPLT) molecules that presented a cooperative response to pH and Ca^2+^. At a high pH value (>9), the surface charge of the nanochannel walls was negative due to the presence of PO_4_^3−^ anions. Subsequently, the nanosystem presented a cation-selective character towards the Ca^2+^ ions that resulted in the neutralisation of the surface charge, i.e., a switch in the polarity of ion transport from cation-selective to non-selective, via which the system turned from the highly conductive to the low-conductive state.

A study by Qu and Tomar [370] employed classical molecular simulation for the study of and insight into thermal diffusivity and thermal conductivity in a set of biomimetic tropocollagen/HA interfaces. Biological principles such as bio-heat transfer are becoming increasingly important in the field of energy storage devices and are being subjected to increasingly intensive scientific research.

## 4. Summary and Concluding Remarks

Nature has always provided inspiration for mankind and it continues to serve as a source and a “treasury” of novel concepts and methods related to the design and development of new materials. In this respect, biomineralisation has been the focus of scientific interest for several decades. The study, understanding, and applications of biomimetic structures and concepts aimed at producing advanced and sophisticated functional materials represent the driving forces behind the rapidly developing “biomimetic/bioinspired material processes” scientific research field.

Researchers have attempted to harness such processes in order to fabricate novel organic/inorganic hybrid materials with fascinating morphologies, outstanding chemical and mechanical properties, and unique biological functions. Biomimetic self-assembly presents a relatively simple technique for the generation of calcium phosphate/protein hybrid materials with controlled morphology and hierarchical structures. Progress in computer science has contributed significantly to the clarification of mineralisation mechanisms and new computational tools enable the exploration of specific recognition mechanisms and the assembly of inorganic/organic interfaces at the nanometric scale in contrast to the possibilities offered by laboratory and clinical studies. The biomechanics of protein/calcium phosphate hybrid structures can now be studied over various time scales with unprecedented degrees of accuracy. Such computer models and simulations are already providing a significant contribution towards enhancing the understanding of the significant problems associated with biomedical engineering.

Initially, the research of biomineralisation processes led to the preparation of common biocomposites with compositions and structures that mimicked real tissue. Over time, however, the development of new methodologies enabled the fabrication of more sophisticated structures such as supramolecular self-assembly nanoreactors and nanoconstructs for “smart” applications e.g., the delivery of multiple biofactors and the sensoring of various biological processes. The continuing search for new alternative protein templates in the natural world, together with the isolation thereof from biowaste, provides almost unlimited possibilities with regard to future research in this area. Although calcium phosphate/protein systems have already been “patented by nature”, they still offer huge potential in the field of biomedical material research.
